# Fatty acid oxidation drives mitochondrial hydrogen peroxide production by α-ketoglutarate dehydrogenase

**DOI:** 10.1016/j.jbc.2024.107159

**Published:** 2024-03-11

**Authors:** Cathryn Grayson, Ben Faerman, Olivia Koufos, Ryan J. Mailloux

**Affiliations:** The School of Human Nutrition, Faculty of Agricultural and Environmental Sciences, McGill University, Quebec, Canada

**Keywords:** alpha-ketoglutarate dehydrogenase, fatty acid oxidation, hydrogen peroxide, mitochondria, oxidative eustress

## Abstract

In the present study, we examined the mitochondrial hydrogen peroxide (mH_2_O_2_) generating capacity of α-ketoglutarate dehydrogenase (KGDH) and compared it to components of the electron transport chain using liver mitochondria isolated from male and female C57BL6N mice. We show for the first time there are some sex dimorphisms in the production of mH_2_O_2_ by electron transport chain complexes I and III when mitochondria are fueled with different substrates. However, in our investigations into these sex effects, we made the unexpected and compelling discovery that 1) KGDH serves as a major mH_2_O_2_ supplier in male and female liver mitochondria and 2) KGDH can form mH_2_O_2_ when liver mitochondria are energized with fatty acids but only when malate is used to prime the Krebs cycle. Surprisingly, 2-keto-3-methylvaleric acid (KMV), a site-specific inhibitor for KGDH, nearly abolished mH_2_O_2_ generation in both male and female liver mitochondria oxidizing palmitoyl-carnitine. KMV inhibited mH_2_O_2_ production in liver mitochondria from male and female mice oxidizing myristoyl-, octanoyl-, or butyryl-carnitine as well. S1QEL 1.1 (S1) and S3QEL 2 (S3), compounds that inhibit reactive oxygen species generation by complexes I and III, respectively, without interfering with OxPhos and respiration, had a negligible effect on the rate of mH_2_O_2_ production when pyruvate or acyl-carnitines were used as fuels. However, inclusion of KMV in reaction mixtures containing S1 and/or S3 almost abolished mH_2_O_2_ generation. Together, our findings suggest KGDH is the main mH_2_O_2_ generator in liver mitochondria, even when fatty acids are used as fuel.

Mitochondrial hydrogen peroxide (mH_2_O_2_) generation in liver tissue is being studied now more than ever due to its role in triggering pathways required to maintain optimal hepatic health. Hepatic mH_2_O_2_ activates nuclear factor erythroid 2–related factor 2, hypoxia inducible factor-1, and heat shock factor-1, which promote antioxidant defense, glycolysis, and protein folding in liver cells (1, 2, 3, 4). It also induces cell cycle proteins like cyclin D to activate cell division in response to liver damage (5). Liver regeneration is also promoted by the mH_2_O_2_-mediated induction of extracellular signal–regulated kinases and protein kinase B (6). mH_2_O_2_ formed in extrahepatic tissues also has many physiological benefits. The genesis of mH_2_O_2_ has been implicated in embryogenesis and tissue development, cell migration, neural activity, wound healing, and many more functions (7, 8, 9). The beneficial signaling effects of mH_2_O_2_ in hepatocytes and many other mammalian cells were recently defined as “oxidative eustress” (10). Oxidative eustress pathways are activated when mH_2_O_2_ occurs in the 1 to 100 nM range and involves the site-specific, rapid, and reversible oxidation of proteinaceous thiols (10, 11). On the other hand, cytotoxic levels of mH_2_O_2_ were recently defined as “oxidative distress.” In this case, oxidative distress occurs when mH_2_O_2_ levels are in the 100 nM cytotoxic range (10, 11).

It is accepted that mH_2_O_2_ can be beneficial or deleterious, which depends on its overall availability. But the source of this mH_2_O_2_ still a matter of debate. Mitochondria can contain up to 12 mH_2_O_2_ sources (12). The 12 generators are classified in two categories based on the redox pair linked to the mH_2_O_2_ production: the NADH/NAD^+^ isopotential group and the CoQH_2_/CoQ isopotential group (13, 14). The first group is comprised of flavin-dependent dehydrogenases that form mH_2_O_2_ in the presence of NAD(H) and consists of α-ketoglutarate dehydrogenase (KGDH), pyruvate dehydrogenase (PDH), branched-chain keto acid dehydrogenase (BCKDH), 2-oxoadipate dehydrogenase (OADH), and the flavin mononucleotide group in the N-module of complex I (9, 12, 15). The CoQH_2_/CoQ isopotential group is composed of *sn*-glycerol-3-phosphate dehydrogenase, proline dehydrogenase, dihydroorotate dehydrogenase (DHODH), electron transferring flavoprotein:coenzyme Q_10_ oxidoreductase, the CoQ binding site in complex I, complex II, and complex III (9, 12, 15). Complex I and III are often credited as the primary sites for mH_2_O_2_ production in the mitochondria of many cell types (16, 17). However, the other potential generators can be significant sources as well under specific (patho)physiological conditions. Proline dehydrogenase is an important mH_2_O_2_ source in insect muscles and cancer cells (18, 19). DHODH is overexpressed in most cancer types where it uses mH_2_O_2_ in signaling (20, 21). Factors like nutrient availability, genetic and epigenetic programming, O_2_ availability, and the concentration of the electron donating center required for mH_2_O_2_ production (*e.g.,* the concentration of the enzyme) impact the overall rate of generation by these individual sources (9, 12, 22, 23, 24).

Empirical evidence collected over the past 2 decades has shown the α-keto acid dehydrogenase family of enzymes, which are part of the NADH/NAD^+^ isopotential group, can produce more mH_2_O_2_ than sources like complex I in many tissues. Additionally, KGDH seems to be the most potent generator out of the four α-keto acid dehydrogenases. KGDH was first documented to be a source of mH_2_O_2_ 2 decades ago when it was found its purified form could generate oxyradicals as a side product during the oxidative decarboxylation of α-ketoglutarate (25). KGDH was shown to be an important mH_2_O_2_ source in rat brain mitochondria and synaptosomes around the same period (26, 27). Since then, a significant amount of data has been collected demonstrating that KGDH displays high rates of mH_2_O_2_ generation in various tissues and cell types. Quinlan *et al*. found KGDH is the most potent mH_2_O_2_ generator in the NADH/NAD^+^ isopotential group in rat muscle mitochondria, displaying a rate of production that is eight times higher than complex I (12, 28). The other members of the NADH/NAD^+^ isopotential group, PDH, BCKDH, and OADH, were also shown by Martin Brand’s group to produce more mH_2_O_2_ than complex I (12, 28, 29, 30). In addition, KGDH accounts for ∼35% of the total mH_2_O_2_ formed by liver mitochondria, whereas complex I generates negligible amounts (31, 32, 33). PDH was identified as a vital source of mH_2_O_2_ in liver and permeabilized muscle fibers of mouse, rat, and human origin as well (31, 32, 33, 34, 35). Purified KGDH from porcine heart can also form large quantities of mH_2_O_2_ during reverse electron transfer (RET) from NADH (26, 36). Recently, it was shown using rodents null for the genes encoding dihydrolipoamide succinyltransferase and dihydrolipoamide dehydrogenase that KGDH can also generate large quantities of mH_2_O_2_ during RET from the electron transport chain (ETC) (37). This observation is important because complex I is viewed as one of the main suppliers of mH_2_O_2_ during RET in the ETC. There are also sex dimorphisms in mH_2_O_2_ by KGDH in liver mitochondria.

Sex is an important biological factor that can also impact the rate of mH_2_O_2_ generation, which can have a profound effect on oxidative eustress signaling in the liver (reviewed here (38, 39)). What is still unknown is how sex can affect the rate of mH_2_O_2_ generation by key sources in hepatic mitochondria, such as KGDH and complexes I and III. The objective of this study was to measure the relative rates of mH_2_O_2_ generation by KGDH and the ETC complexes I and III in male and female liver mitochondria to ascertain the effect of sex on these main hepatic generators. Using a substrate/inhibitor toolkit that we have developed, we show there are fundamental sex differences in mH_2_O_2_ generation by the individual complexes and that female liver mitochondria, in general, produce less mH_2_O_2_ due to greater OxPhos efficiency (9). However, in this study, we made the unexpected finding that 1) KGDH is an important source of mH_2_O_2_ in liver mitochondria from male and female mice oxidizing fatty acids and 2) KGDH is the main mH_2_O_2_ generator in liver mitochondria, not complex I or complex III. The implications of these findings and how they apply to liver physiology and the progression of hepatic diseases are discussed herein.

## Results

### Female liver mitochondria from C57BL6N mice produce less mH_2_O_2_ due to greater OxPhos efficiency

**Figure 1:**
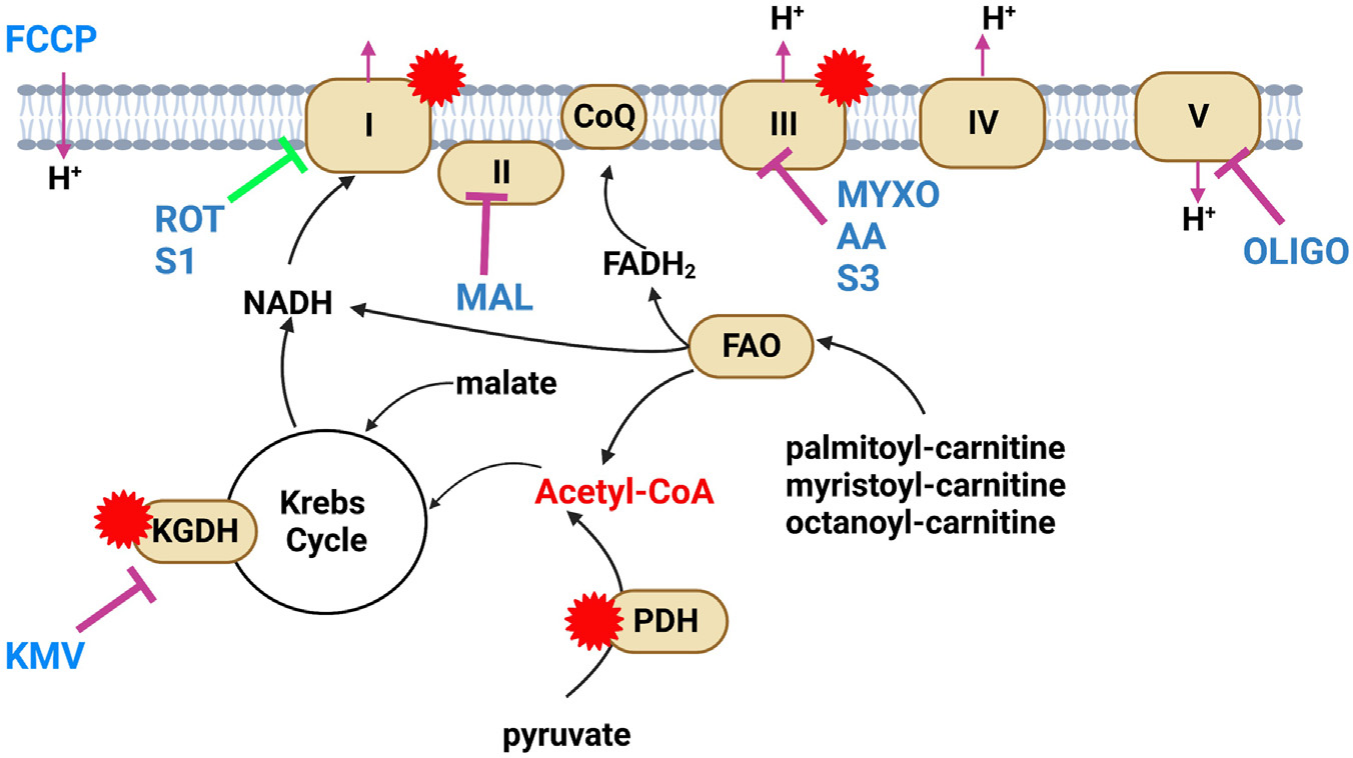
**Depiction of the experimental design for interrogating mH**_**2**_**O**_**2**_**generation by α-ketoglutarate dehydrogenase and the individual components of the electron transport chain, complexes I, II, and III.** Inhibitor and substrate combinations used for this study are listed in the figure. Reaction mixtures consisted of combinations of pyruvate, succinate, and fatty acyl-carnitines of variable chain length. Malate was added in reactions containing pyruvate or fatty acids to prime the Krebs cycle. Inhibitors used include 2-keto-3-methylvaleric acid (KMV; KGDH), rotenone and S1QEL 1.2 (Rot, S1; complex I), malonate (Mal; complex II), myxothiazol, antimycin A, and S3QEL 2 (Myxo, AA, S3; complex III), and oligomycin (Oligo; complex V). FCCP was used in the XFe24 assays to estimate the maximal rate of respiration in mitochondria. Different substrates used to power mitochondria for mH_2_O_2_ measurements and O_2_ consumption assays are listed in the figure (pyruvate, malate, succinate, and various fatty acyl-carnitines). The points at which each substrate feeds into nutrient oxidation pathways for mH_2_O_2_ generation and OxPhos are shown. The sources of mH_2_O_2_ and the orientation of production relative to the inner mitochondrial membrane (IMM) are shown as a *red star*. KMV, 2-keto-3-methylvaleric acid; mH_2_O_2_, mitochondrial hydrogen peroxide.

**Figure 2:**
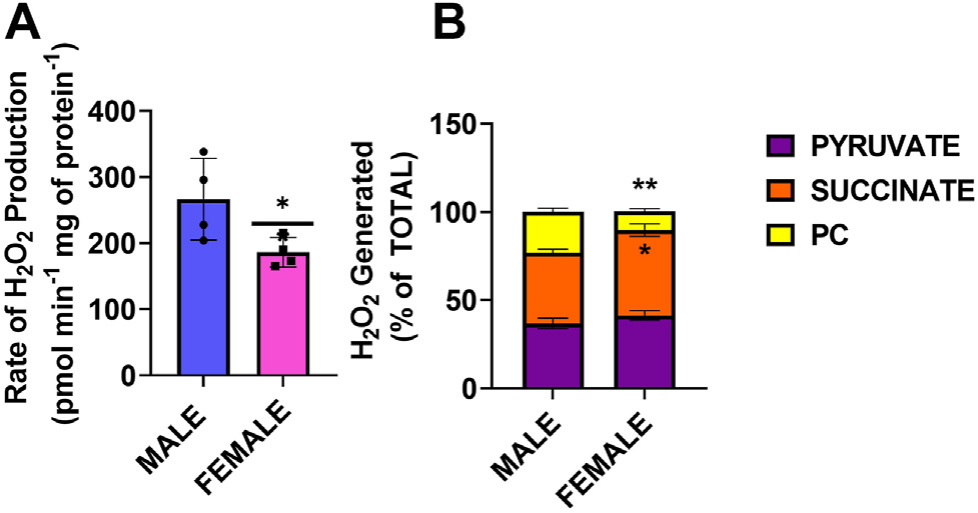
**Female liver mitochondria generate less mH**_**2**_**O**_**2**_**when compared to male samples.***A*, the rates for mH_2_O_2_ generation by male and female liver mitochondria–oxidizing pyruvate, succinate, and palmitoyl-carnitine with malate. n = 4, mean ± SD, two-tailed Student’s *t* test. *B*, the percent contributions of the different substrates toward the total rate of mH_2_O_2_ production by male and female liver mitochondria. n = 4, mean ± SD, two-tailed Student’s *t* test. mH_2_O_2_, mitochondrial hydrogen peroxide.

Figure 1 provides an experimental design summary of the substrate and inhibitor combinations used to probe the relative rate of mH_2_O_2_ production and the states of respiration in isolated liver mitochondria. First, we examined the effect of sex on mH_2_O_2_ generation by liver mitochondria oxidizing a combination of pyruvate, succinate, and palmitoyl-carnitine with malate to prime the Krebs cycle (Fig. 2*A*). The rate of mH_2_O_2_ generation was significantly lower in female liver mitochondria than in males (Fig. 2*A*). Examining the individual contributions of the different substrates toward total mH_2_O_2_ generation revealed sex differences in the use of succinate and palmitoyl-carnitine in the production of mH_2_O_2_. Female liver mitochondria used significantly more succinate for the generation of mH_2_O_2_ (Fig. 2*B*). Palmitoyl-carnitine was a more potent substrate for mH_2_O_2_ generation in male liver mitochondria when compared to female samples (Fig. 2*B*). No significant differences in the percent contribution of pyruvate toward the rate of mH_2_O_2_ production were observed between male and female liver mitochondria (Fig. 2*B*).

Next, we interrogated the rates of oxygen consumption under different states of respiration to discern if the lower rate of mH_2_O_2_ generation in the female liver mitochondria was due to the better coupling of nutrient oxidation to OxPhos. First, we found no differences in OCR under state 4, state 3, state 4_O_, or state 3_U_ respiratory conditions in the male liver mitochondria energized with pyruvate and malate, succinate, or palmitoyl-carnitine and malate, respectively (Fig. S1). No substrate-dependent differences were observed in female liver mitochondria as well except when succinate was fueling state 3_U_ respiration (Fig. S1).

**Figure 3:**
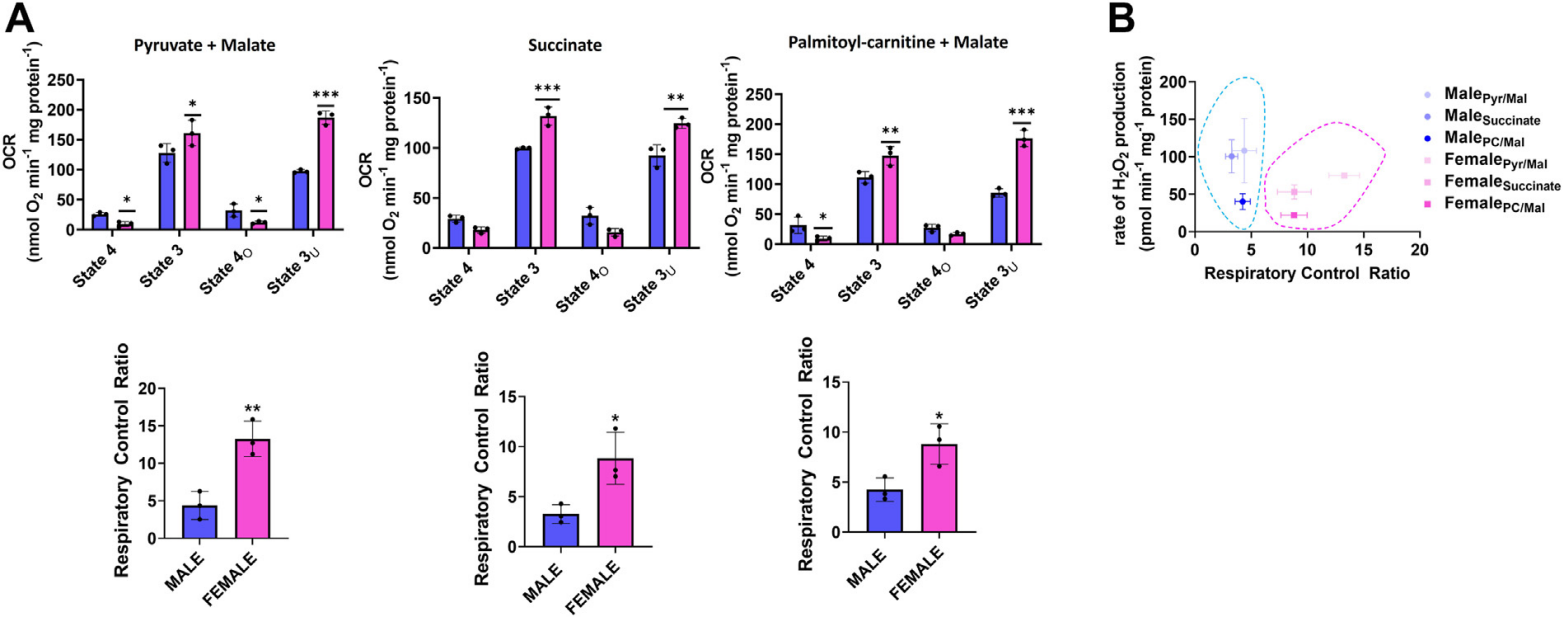
**The decreased rate of mH**_**2**_**O**_**2**_**production correlates with more efficient nutrient oxidation and OxPhos in female liver mitochondria.***A*, sequential measurement of rates of O_2_ consumption under state 4, state 3, state 4_O_, and state 3_U_ by male and female liver mitochondria oxidizing different substrates. State 4 was measured first by the addition of the substrate(s). State 3, state 4_O_, and state 3_U_ respiration were then induced by the sequential addition of ADP, oligomycin, and FCCP, respectively. All values were corrected for O_2_ consumption recorded after the addition of antimycin A (to arrest the ETC). The values for state 3 and state 4_O_ were used to calculate the respiratory control ratio (RCR) under each condition. n = 3, mean ± SD, two-tailed Student’s *t* test. *B*, the mitochondria used in the Seahorse XFe24 assays were also used measure the rate of mH_2_O_2_ generation, which was then plotted as a function of the RCR. ETC, electron transport chain; mH_2_O_2_, mitochondrial hydrogen peroxide.

Comparing the OCR of male and female liver mitochondria–oxidizing pyruvate and malate, succinate, or palmitoyl-carnitine and malate revealed a sex effect in the efficiency of respiration. Nonphosphorylating OCR (proton leak–dependent or state 4) was slightly higher in the male liver mitochondria and was significant in the cases of samples oxidizing pyruvate and malate or palmitoyl-carnitine and malate (Fig. 3*A*). Liver mitochondria from female mice displayed a significantly greater OCR under state 3 (ADP-stimulated) conditions when energized with pyruvate and malate, succinate, or palmitoyl-carnitine and malate, respectively (Fig. 3*A*). State 4_O_, proton leak–dependent respiration measured in the presence of oligomycin, was only significantly higher in male liver mitochondria–oxidizing pyruvate and malate (Fig. 3*A*). Measurement of state 3_U_, or OCR induced by the protonophore FCCP, revealed max OCR was significantly greater in female liver mitochondria oxidizing either of the three substrate combinations (Fig. 3*A*). Calculation of the respiratory control ratio (RCR), a proxy measure for the efficiency of OxPhos, showed it was ∼2-fold higher in females than in males in mitochondria-oxidizing succinate or palmitoyl-carnitine with malate and ∼2.5-fold greater in female samples energized with pyruvate and malate (Fig. 3*A*). Plotting the rate of mH_2_O_2_ generation by male and female liver mitochondria–oxidizing pyruvate and malate, succinate, or palmitoyl-carnitine and malate against the RCR of these samples revealed that the lower rate of mH_2_O_2_ generation by female liver mitochondria correlated with greater RCR values (Fig. 3*B*). By contrast, the higher rate of mH_2_O_2_ generation in the male liver mitochondria under all substrate conditions was related to the lower RCR (Fig. 3*B*).

**Figure 4:**
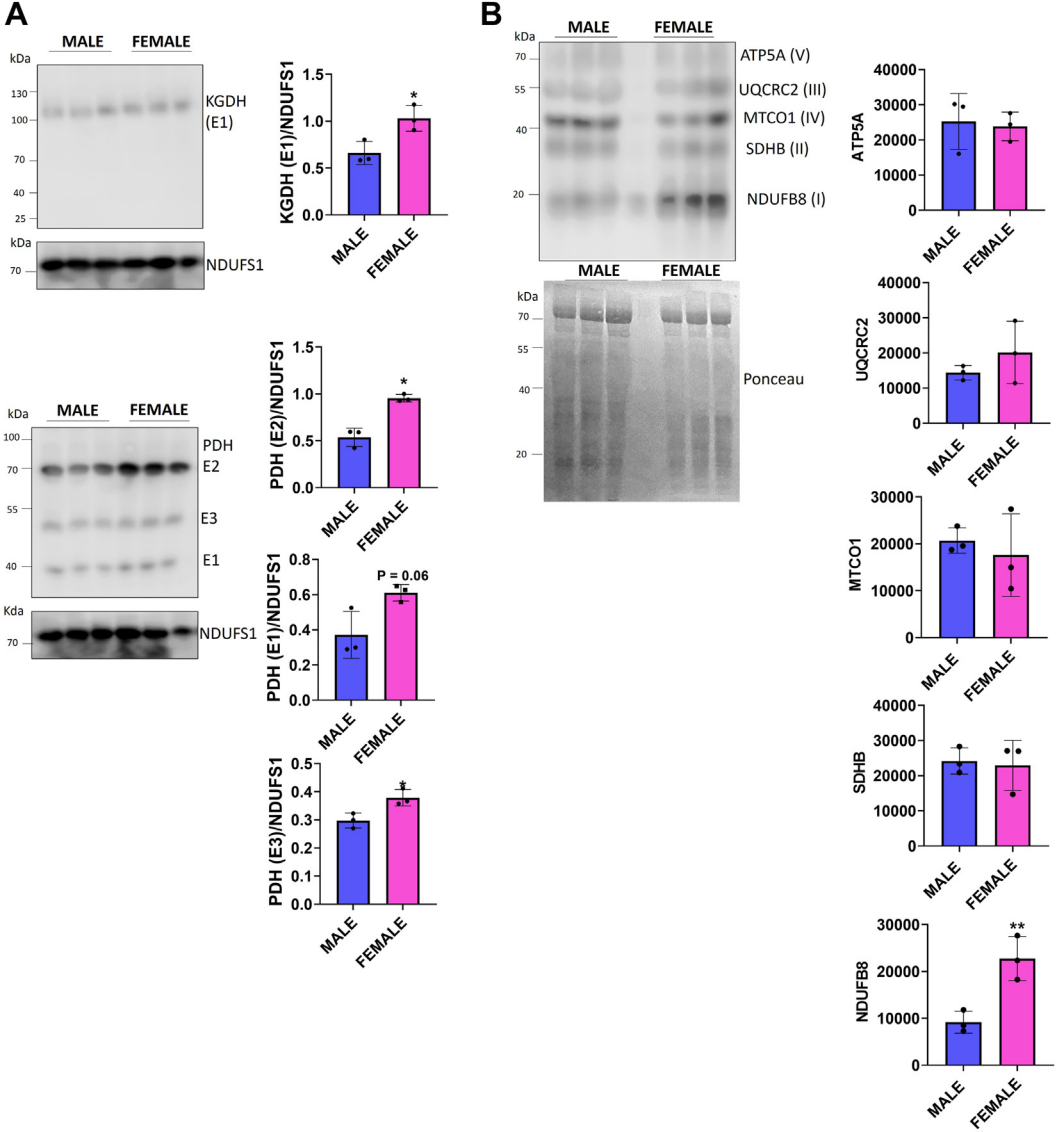
**The greater OxPhos efficiency and lower rate for mH**_**2**_**O**_**2**_**generation in female liver mitochondria is related to the higher protein expression of PDH, KGDH, and complex I.***A*, immunoblot analysis of the E2 subunit of KGDH and the E1 to E3 subunits of PDH. NDUFS1 for complex I was used as the loading control. n = 3, mean ± SD, two-tailed Student’s *t* test. Blots were quantified with ImageJ. *B*, immunoblot analysis of the expression of the respiratory complexes I to V using the OxPhos antibody cocktail. The subunits that correspond with the individual complexes are indicated in the *brackets* in the figure. n = 3, mean ± SD, two-tailed Student’s *t* test. Blots were quantified with ImageJ. ETC, electron transport chain; KGDH, α-ketoglutarate dehydrogenase; mH_2_O_2_, mitochondrial hydrogen peroxide; PDH, pyruvate dehydrogenase.

Expression of the E1 subunit for KGDH was higher in the female liver mitochondria than in males (Fig. 4*A*). The three PDH subunits were also significantly higher in protein level than in male liver mitochondria (Fig. 4*A*). Use of the OxPhos antibody cocktail revealed no significant differences in SdhB (complex II), UQCRC2 (complex III), MTCO1 (complex IV), or ATP5A (complex V) levels (Fig. 4*B*). However, the abundance of NDUFB8 (complex I) was ∼2-fold greater in female hepatic mitochondria (Fig. 4*B*). In aggregate, male and female liver mitochondria display a sex difference in mH_2_O_2_ generation, which is characterized by a decreased overall rate in mH_2_O_2_ production by female samples. This decreased mH_2_O_2_ generation was attributed to the increased efficiency of OxPhos in the female liver mitochondria.

### KGDH is a major mH_2_O_2_ source in male and female liver mitochondria

The results collected in Figure 2 showed succinate and palmitoyl-carnitine are used differently by liver mitochondria from male and female C57BL6N mice to drive mH_2_O_2_ generation, something that was not observed with pyruvate. Notably, both succinate and palmitoyl-carnitine are oxidized directly by the ETC, whereas pyruvate must first enter the Krebs cycle before it can be used for OxPhos. Pyruvate drives mH_2_O_2_ generation by the ETC, but also through PDH and KGDH. We have demonstrated before that KGDH and complex III are the main mH_2_O_2_ generators during the mitochondrial oxidation of pyruvate, lactate, or α-ketoglutarate in liver mitochondria from male mice (31, 32, 40). Together, with our findings in Figure 2, we decided to interrogate the individual rates of mH_2_O_2_ generation by KGDH and the complexes in the ETC using the substrate and inhibitor combinations depicted in Figure 1.

**Figure 5:**
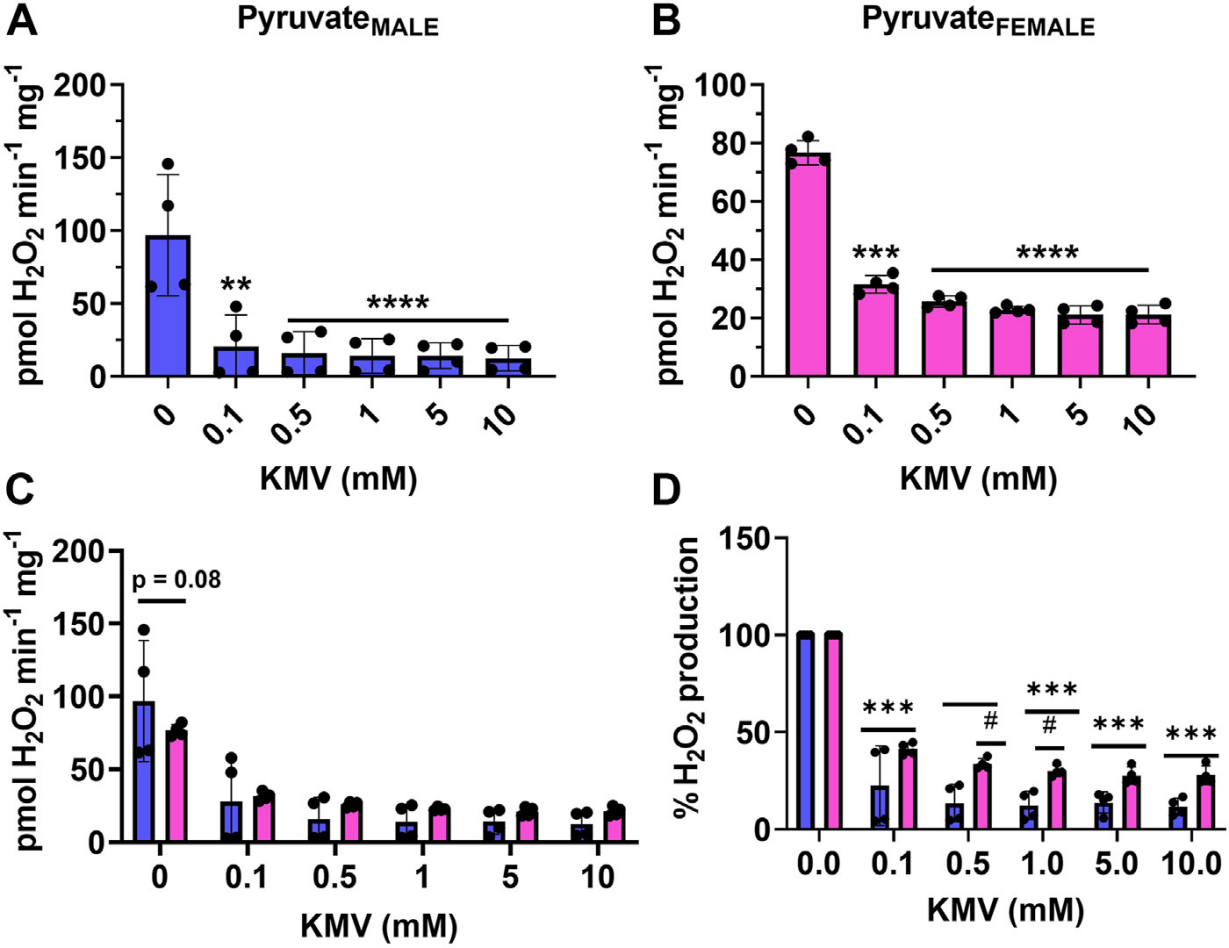
**KGDH is a major mH**_**2**_**O**_**2**_**supplier in liver mitochondria–oxidizing pyruvate and malate.***A*, the effect of increasing doses of competitive and site-specific inhibitor for KGDH, KMV, on the rate of mH_2_O_2_ generation by male liver mitochondria–oxidizing pyruvate and malate. n = 4, mean ± SD, one-way ANOVA with a post hoc Fisher’s LSD test. *B*, the effect of increasing doses of competitive and site-specific inhibitor for KGDH, KMV, on the rate of mH_2_O_2_ generation by female liver mitochondria–oxidizing pyruvate and malate. n = 4, mean ± SD, one-way ANOVA with a post hoc Fisher’s LSD test. *C*, comparison of the rates of mH_2_O_2_ production by male and female liver mitochondria energized with pyruvate and malate and incubated in increasing doses of KMV. n = 4, mean ± SD, two-way ANOVA with a post hoc Fisher’s LSD test. *D*, calculation of the percent change in the rate of mH_2_O_2_ production relative to control in response to increasing doses of KMV. n = 4, mean ± SD, two-way ANOVA with a post hoc Fisher’s LSD test. ∗ denotes a dose-dependent effect in the male or female mitochondria, and # denotes a sex effect at the different doses. KGDH, α-ketoglutarate dehydrogenase; KMV, 2-keto-3-methylvaleric acid; mH_2_O_2_, mitochondrial hydrogen peroxide.

Our titrations with the KGDH inhibitor, KMV, revealed that it is a major source of mH_2_O_2_ generation in male and female liver mitochondria–oxidizing pyruvate in the presence of malate (Fig. 5, *A* and *B*). KMV is a competitive inhibitor for KGDH, and it was previously reported that 10 mM is required to induce maximum inhibition of the enzyme (28). In our hands we were able to show that maximal inhibition of mH_2_O_2_ production with KMV in mitochondria–oxidizing pyruvate and malate can be achieved with as little as 0.1 mM (Fig. 5). The KMV would also hamper the downstream production of mH_2_O_2_ by the ETC as well which may account for the abolishment of production at such a low concentration (Fig. 5). Comparison of the rates of mH_2_O_2_ production by the male and female liver mitochondria–oxidizing pyruvate and malate revealed sex influences production under these conditions. Indeed, the rate of mH_2_O_2_ genesis was lower in the female samples than in the males (Fig. 5*C*). However, the rate of mH_2_O_2_ generation was nearly abolished at all KMV concentrations in the male and female liver mitochondria–oxidizing pyruvate and malate (Fig. 5*C*). Indeed, KMV at a concentration of 0.1 mM decreased mH_2_O_2_ genesis in the male and female samples by ∼90% (indicated by asterisks) (Fig. 5*D*). Furthermore, this KMV effect (∼90% suppression of mH_2_O_2_ production) persisted at higher inhibitor concentrations (Fig. 5*D*). We observed no sex differences in the percent mH_2_O_2_ production relative to control in the male and female liver mitochondria treated with the different KMV concentrations (Fig. 5*D*).

### Sex affects mH_2_O_2_ generation by the ETC in a substrate-specific manner

**Figure 6:**
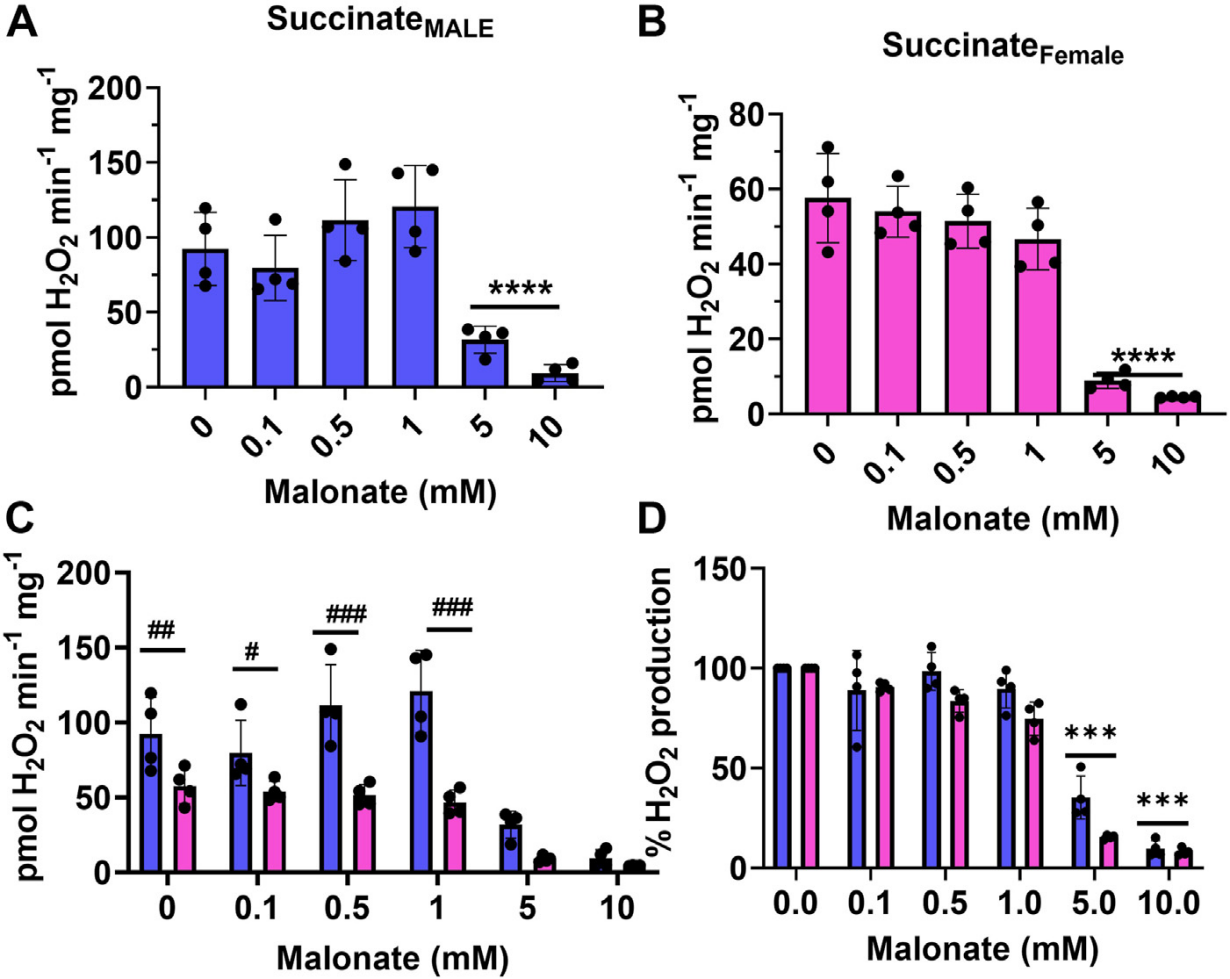
**There is no sex dimorphic effect in the response of succinate-driven mH**_**2**_**O**_**2**_**production in response to malonate treatment.***A*, impact of competitive and site-specific inhibitor for complex II, malonate, on the rate of mH_2_O_2_ generation by male liver mitochondria–oxidizing succinate. n = 4, mean ± SD, one-way ANOVA with a post hoc Fisher’s LSD test. *B*, the effect of increasing doses of competitive and site-specific inhibitor for complex II, malonate, on the rate of mH_2_O_2_ generation by female liver mitochondria–oxidizing succinate. n = 4, mean ± SD, one-way ANOVA with a post hoc Fisher’s LSD test. *C*, comparison of the rates of mH_2_O_2_ production by male and female liver mitochondria energized with succinate in response to increasing doses of malonate. n = 4, mean ± SD, two-way ANOVA with a post hoc Fisher’s LSD test. *D*, calculation of the percent change in the rate of mH_2_O_2_ production relative to control in response to increasing doses of malonate. n = 4, mean ± SD, two-way ANOVA with a post hoc Fisher’s LSD test. ∗ denotes a dose-dependent effect in the male or female mitochondria, and # denotes a sex effect at the different doses. mH_2_O_2_, mitochondrial hydrogen peroxide.

Next, we examined mH_2_O_2_ production during succinate metabolism. For this, all samples were supplied with rotenone to inhibit mH_2_O_2_ generation by complex I through RET from succinate. This allows the indirect determination of whether complex I is a mH_2_O_2_ source after RET from succinate. Figure 6*A* shows the effect of malonate, a competitive inhibitor that interferes with the succinate binding site in the SdhA subunit of complex II, on mH_2_O_2_ in male and female liver mitochondria. Supplying mitochondria with malonate at a concentration lower than the amount of succinate (5 mM) had no significant effect on the rate of mH_2_O_2_ generation in liver mitochondria from both male and female mice (Fig. 6, *A* and *B*). Adding malonate to a concentration that was equal to that of succinate (5 mM) strongly inhibited mH_2_O_2_ generation in male and female liver mitochondria (Fig. 6, *A* and *B*). mH_2_O_2_ generation was abolished at 10 mM malonate. Rate comparisons revealed female hepatic mitochondria produced significantly less mH_2_O_2_ than male counterparts (Fig. 6*C*). This is interesting considering we found in Fig. 2 that succinate oxidation made a greater contribution to the total percentage of the mH_2_O_2_ formed by female liver mitochondria. Despite this difference in substrate preference for mH_2_O_2_ generation, the absolute rate for production during succinate oxidation was still significantly lower in the female liver mitochondria than in males (Fig. 6*C*). Calculation of the percent inhibition of mH_2_O_2_ generation revealed malonate had little to no effect on the rate of generation when mitochondria from male and female livers were treated with ≤1 mM (Fig. 6*D*). However, concentrations of malonate ≥5 mM almost completely abolished mH_2_O_2_ generation by mitochondria from the livers of male and female mice (asterisks) (Fig. 6*D*). However, no sex effects were observed at the individual concentrations of malonate, indicating male and female liver mitochondria are equally sensitive toward the malonate-mediated deactivation of succinate-driven mH_2_O_2_ formation (Fig. 6*D*).

**Figure 7:**
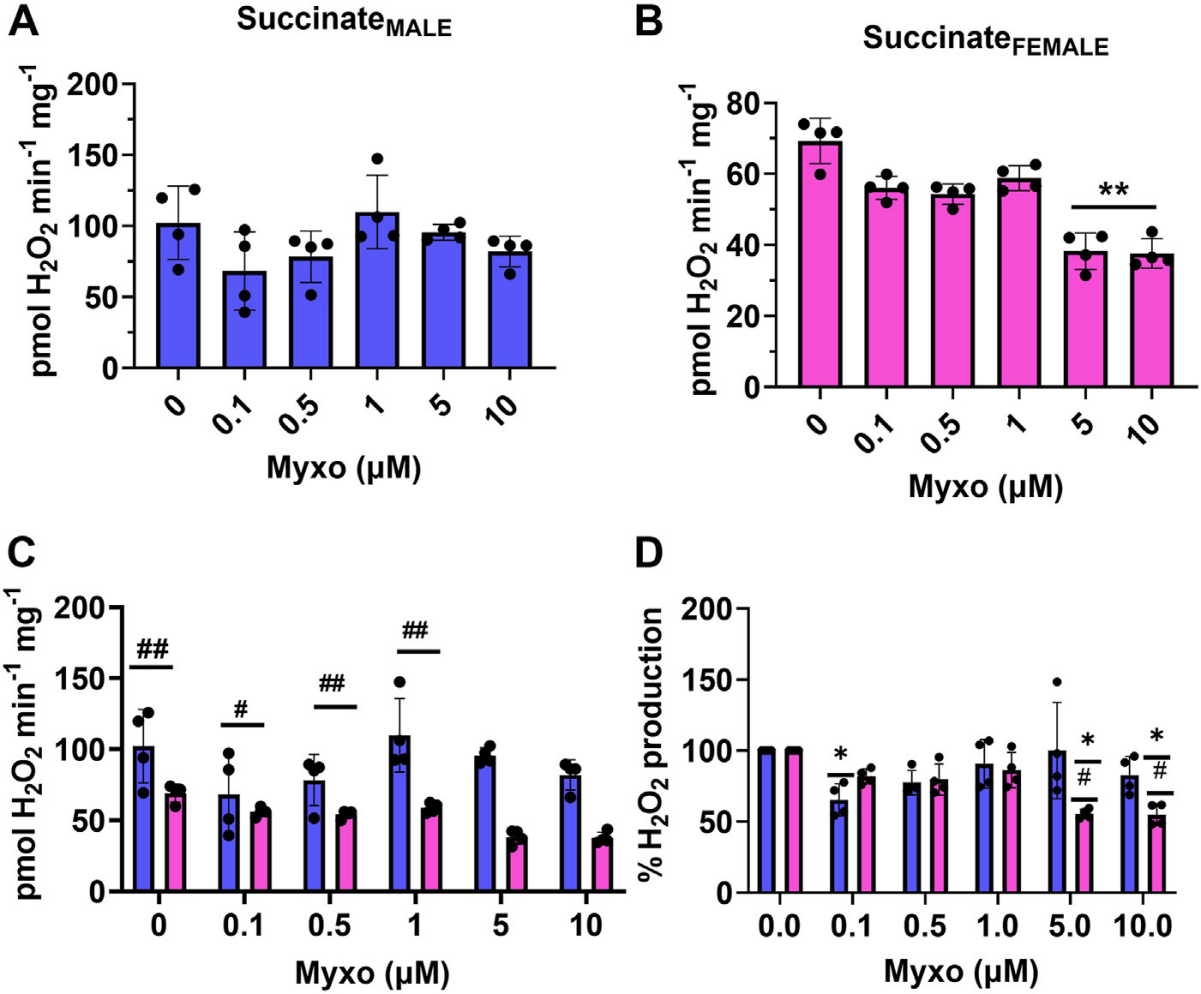
**Myxothiazol inhibits succinate-induced mH**_**2**_**O**_**2**_**production by female, but not male, liver mitochondria.***A*, measurement of mH_2_O_2_ generation by male liver mitochondria–oxidizing succinate in response to increasing doses of myxothiazol. n = 4, mean ± SD, one-way ANOVA with a post hoc Fisher’s LSD test. *B*, measurement of mH_2_O_2_ generation by female liver mitochondria–oxidizing succinate in response to increasing doses of myxothiazol. n = 4, mean ± SD, one-way ANOVA with a post hoc Fisher’s LSD test. *C*, comparison of the rates of mH_2_O_2_ production by male and female liver mitochondria–metabolizing succinate and treated with myxothiazol. n = 4, mean ± SD, two-way ANOVA with a post hoc Fisher’s LSD test. *D*, calculation of the percent change in the rate of mH_2_O_2_ production relative to control in response to increasing doses of myxothiazol. n = 4, mean ± SD, one-way ANOVA with a post hoc Fisher’s LSD test. ∗ denotes a dose-dependent effect in the male or female mitochondria, and # denotes a sex effect at the different doses. mH_2_O_2_, mitochondrial hydrogen peroxide.

We also examined the impact of myxothiazol on succinate-driven mH_2_O_2_ generation (Fig. 7). Myxothiazol inhibits superoxide (O_2_^•-^) formation by complex III *via* limitation of semiquinone radical production in its ubiquinol binding site. Thus, myxothiazol is a valuable tool in quantifying the rate of complex III–driven mH_2_O_2_ generation. We also included rotenone to limit RET to complex I. Surprisingly, titrating myxothiazol into reaction chambers did not affect mH_2_O_2_ generation by male liver mitochondria–oxidizing succinate (Fig. 7*A*). Taken with the results collected with malonate in Figure 6, this would imply complex I is a major source of mH_2_O_2_ generation during succinate metabolism in male liver mitochondria. However, at 5 and 10 μM, myxothiazol induced a ∼45% decrease in mH_2_O_2_ generation by female liver mitochondria (Fig. 7*B*). Thus, given the conditions we have imposed in the experiment, this result indicates complex III, rather than complex I, is an important mH_2_O_2_ generator in female liver mitochondria energized with succinate. Comparison of absolute rates of mH_2_O_2_ production revealed, again, that female mitochondria generated less mH_2_O_2_ when metabolizing succinate, even when myxothiazol was titrated into the reaction chambers (Fig. 7*C*). We also calculated the percent inhibition of mH_2_O_2_ production by myxothiazol in male and female liver mitochondria energized with succinate (Fig. 7*D*). We found treatment with 0.1 mM myxothiazol did induce a small but significant decrease in the mH_2_O_2_ generation rate in male liver mitochondria (asterisk) (Fig. 7*D*). However, no significant effects in the male liver mitochondria were observed at higher myxothiazol concentrations (Fig. 7*D*). No significant effects were observed in the female mice treated with ≤1 μM myxothiazol (Fig. 7*D*). Increasing the concentration of myxothiazol to ≥5 μM resulted in the significant inhibition of mH_2_O_2_ generation by the female liver mitochondria (asterisks) (Fig. 7*D*). This inhibition was also sex-dependent as the myxothiazol (≥5 μM) significantly lowered the percentage of mH_2_O_2_ production relative to the control in the female liver mitochondria than the males (hashtag) (Fig. 7*D*).

**Figure 8:**
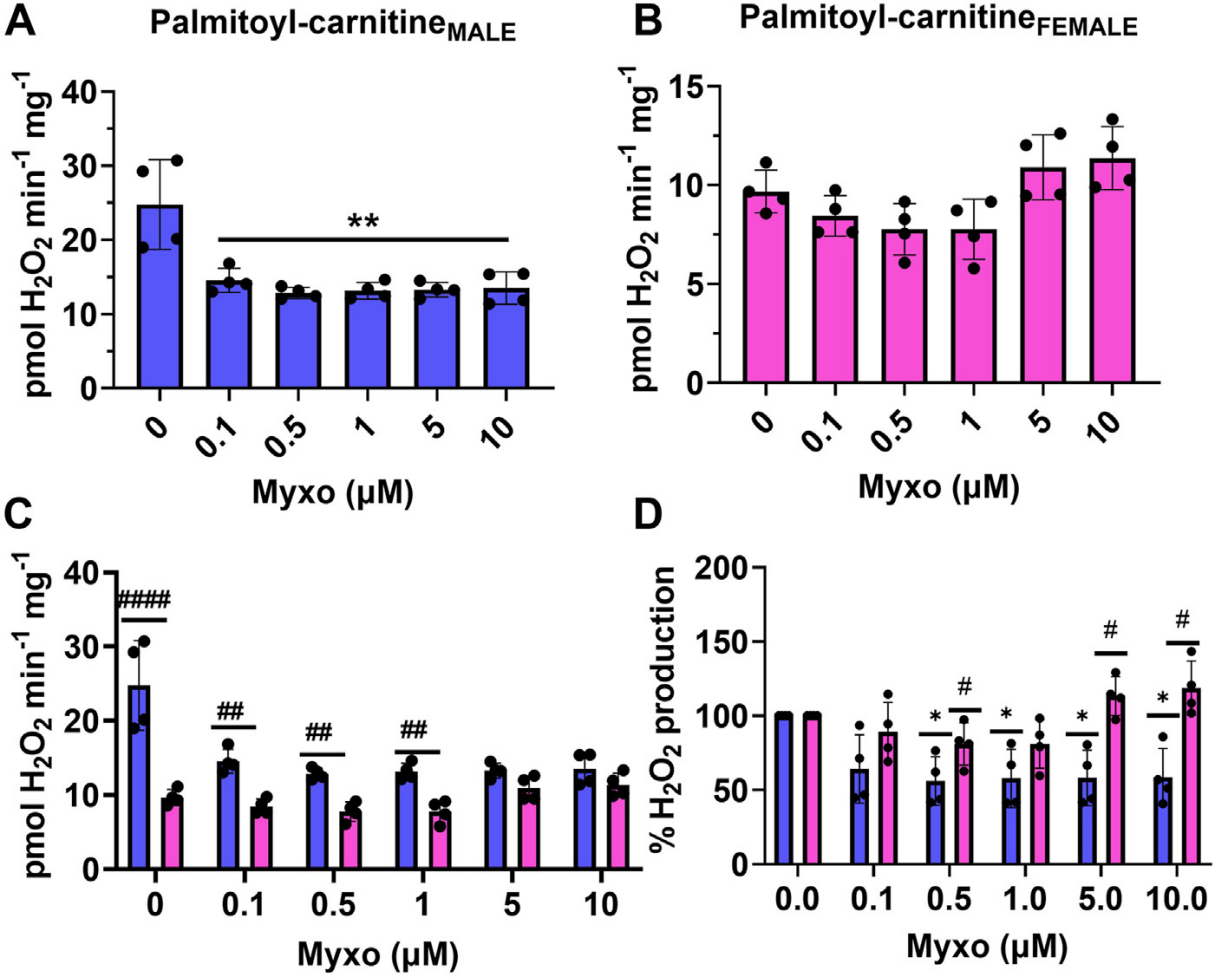
**Myxothiazol inhibits palmitoyl-carnitine-induced mH**_**2**_**O**_**2**_**production by male, but not female, liver mitochondria.***A*, measurement of the rate of mH_2_O_2_ production by male liver mitochondria–oxidizing palmitoyl-carnitine in the presence of malate and in response to increasing doses of myxothiazol. n = 4, mean ± SD, one-way ANOVA with a post hoc Fisher’s LSD test. *B*, measurement of the rate of mH_2_O_2_ production by female liver mitochondria–oxidizing palmitoyl-carnitine in the presence of malate and in response to increasing doses of myxothiazol. n = 4, mean ± SD, one-way ANOVA with a post hoc Fisher’s LSD test. *C*, comparison of the rates of mH_2_O_2_ production by male and female liver mitochondria energized with palmitoyl-carnitine and malate and treated with myxothiazol. n = 4, mean ± SD, two-way ANOVA with a post hoc Fisher’s LSD test. *D*, calculation of the percent change in the rate of mH_2_O_2_ production relative to control in response to increasing doses of myxothiazol. n = 4, mean ± SD, two-way ANOVA with a post hoc Fisher’s LSD test. ∗ denotes a dose-dependent effect in the male or female mitochondria, and # denotes a sex effect at the different doses. mH_2_O_2_, mitochondrial hydrogen peroxide.

Next, we examined mH_2_O_2_ generation in mitochondria metabolizing palmitoyl-carnitine and malate. Previous studies established palmitoyl-carnitine (in the absence of malate) generates most of its mH_2_O_2_ through the ETC. Rotenone was added to inhibit mH_2_O_2_ production by RET to complex I. At 0.1 μM, myxothiazol inhibited mH_2_O_2_ generation by male liver mitochondria–oxidizing palmitoyl-carnitine and malate (Fig. 8*A*). This inhibition remained constant at higher myxothiazol concentrations. No myxothiazol effect was observed in female liver mitochondria–oxidizing palmitoyl-carnitine and malate (Fig. 8*B*). The rate of mH_2_O_2_ generation was significantly higher in male liver mitochondria–oxidizing palmitoyl-carnitine and malate than in female samples (Fig. 8*C*). Examination of the inhibitory effect of myxothiazol as a percentage of the rate of mH_2_O_2_ production in control samples revealed it inhibited mH_2_O_2_ generation in male liver mitochondria by ∼45% at 0.1 μM (asterisks) (Fig. 8*D*). This significant inhibition also occurred at higher myxothiazol concentrations in the male, but not female, liver mitochondria (asterisks) (Fig. 8*D*). Additionally, there was a sex effect in terms of the response of the male and female liver mitochondria toward the individual concentrations of myxothiazol (hashtag) (Fig. 8*D*). Indeed, myxothiazol significantly lowered mH_2_O_2_ generation in the male liver mitochondria at concentration ≥0.5 μM than in female samples exposed to the same dose (Fig. 8*D*).

In aggregate, using various substrate and inhibitor combinations, we were able to show the following in Figure 5, Figure 6, Figure 7, Figure 8: 1) KGDH is a major source of mH_2_O_2_ in both the male and female liver mitochondria during pyruvate metabolism, 2) succinate metabolism drives mH_2_O_2_ formation through RET to complex I in male, but not female, liver mitochondria, 3) complex III is a source of mH_2_O_2_ when succinate is oxidized by female, but not male, liver mitochondria, 4) complex I supplied mH_2_O_2_ by RET when palmitoyl-carnitine (with malate) is oxidized by female liver mitochondria only, 5) complex III is a mH_2_O_2_ source during palmitoyl-carnitine (with malate) metabolism in male liver mitochondria, 6) although female mitochondria, in general, produce less mH_2_O_2_ when compared to males, sex affects which component of the ETC produces mH_2_O_2_, which is dependent on substrate type, and 7) we reveal for the first time a sex-dependent response toward mitochondrial poisons that are used in the interrogation of mH_2_O_2_ production, namely, myxothiazol. No sex effects for responses toward KMV or malonate were observed.

### KGDH is a major source of mH_2_O_2_ during palmitoyl-carnitine oxidation

The oxidation of fatty acyl-carnitines by the ETC generates acetyl-CoA, which undergoes further oxidation in the Krebs cycle after it condenses with oxaloacetate. This would imply acetyl groups originating from fatty acid oxidation (FAO) pathways can generate mH_2_O_2_ by KGDH when oxaloacetate is present. For the most part, measurement of mH_2_O_2_ generation during FAO in isolated mitochondria is typically conducted in the absence of malate. This is because it is assumed that the bulk of the mH_2_O_2_ generated during FAO originates from the ETC. Our findings in Figure 8 did show myxothiazol inhibited mH_2_O_2_ generation during palmitoyl-carnitine oxidation in liver mitochondria from male mice. No myxothiazol effect was observed in samples collected from female mice (Fig. 8). Notably, malate was included in these reaction mixtures, implying the source of mH_2_O_2_ generation during FAO may be outside of the ETC. Based on this and the results collected in Figure 5 and in our previous studies, we reasoned the main source of mH_2_O_2_ production by liver mitochondria–oxidizing palmitoyl-carnitine and malate was KGDH.

**Figure 9:**
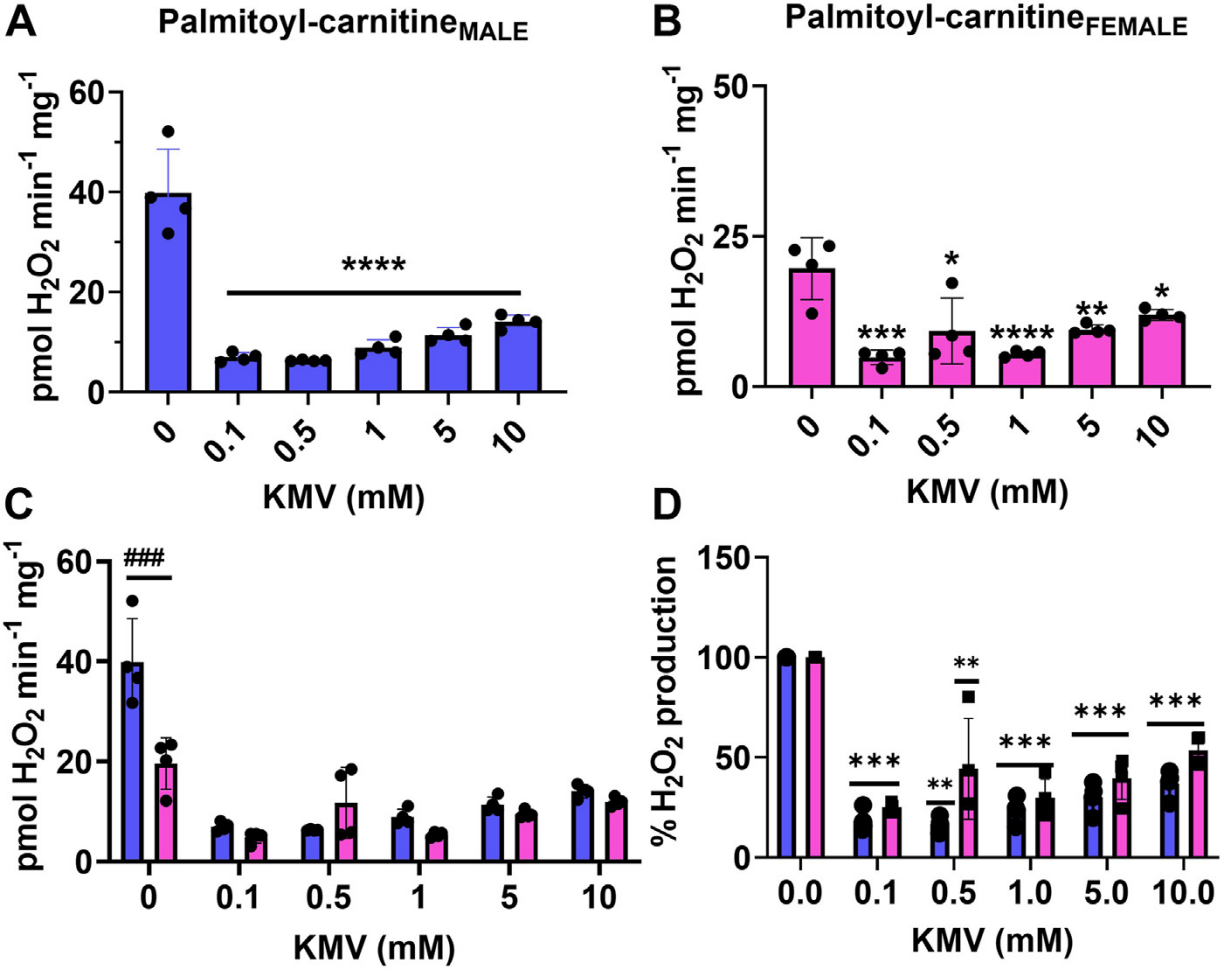
**KGDH is a main source of mH**_**2**_**O**_**2**_**generation during the oxidation of palmitoyl-carnitine in male and female liver mitochondria.***A*, the effect of increasing doses of KGDH inhibitor KMV on the rate of mH_2_O_2_ generation by male liver mitochondria–oxidizing palmitoyl-carnitine and malate. n = 4, mean ± SD, one-way ANOVA with a post hoc Fisher’s LSD test. *B*, the effect of increasing doses of KGDH inhibitor KMV on the rate of mH_2_O_2_ generation by male liver mitochondria–oxidizing palmitoyl-carnitine and malate. n = 4, mean ± SD, one-way ANOVA with a post hoc Fisher’s LSD test. *C*, comparison of the rates of mH_2_O_2_ production by male and female liver mitochondria energized with palmitoyl-carnitine and malate and incubated in increasing doses of KMV. n = 4, mean ± SD, two-way ANOVA with a post hoc Fisher’s LSD test. *D*, calculation of the percent change in the rate of mH_2_O_2_ production relative to control in response to increasing doses of KMV. n = 4, mean ± SD, two-way ANOVA with a post hoc Fisher’s LSD test. ∗ denotes a dose-dependent effect in the male or female mitochondria, and # denotes a sex effect at the different doses. KGDH, α-ketoglutarate dehydrogenase; KMV, 2-keto-3-methylvaleric acid; mH_2_O_2_, mitochondrial hydrogen peroxide.

To test this, we supplied mitochondria with palmitoyl-carnitine and malate and then titrated the site-specific KGDH inhibitor, KMV, into the reaction chambers. Figure 9*A* shows KMV almost abolished mH_2_O_2_ generation when male liver mitochondria were oxidizing palmitoyl-carnitine in the presence of malate. A similar observation was made with the female liver mitochondria (Fig. 9*B*). Adding KMV to a concentration as low as 0.1 mM induced a significant decrease in mH_2_O_2_ generation (Fig. 9*B*). Notably, in males and females, KMV retained its inhibitory effect at all concentrations used—even though some variability was detected, especially in the female samples (Fig. 9, *A* and *B*). The mH_2_O_2_ generation rate in mitochondria fueled with palmitoyl-carnitine and malate was lower in the female liver samples (Fig. 9*C*). We also calculated the percent inhibition of mH_2_O_2_ generation by mitochondria–oxidizing palmitoyl-carnitine and malate exposed to the KMV compound (Fig. 9*D*). In the male mitochondria, KMV as low as 0.1 mM almost completely abolished mH_2_O_2_ generation, an effect that persisted at much higher concentrations and fluctuating at ∼80 to 90% inhibition (asterisks) (Fig. 9*D*). We observed similar KMV effects in the female liver mitochondria, with KMV inducing an ∼80 to 90% inhibition of mH_2_O_2_ generation at all concentrations (Fig. 9*D*). No sex effects were observed between the males and females at all KMV doses.

**Figure 10:**
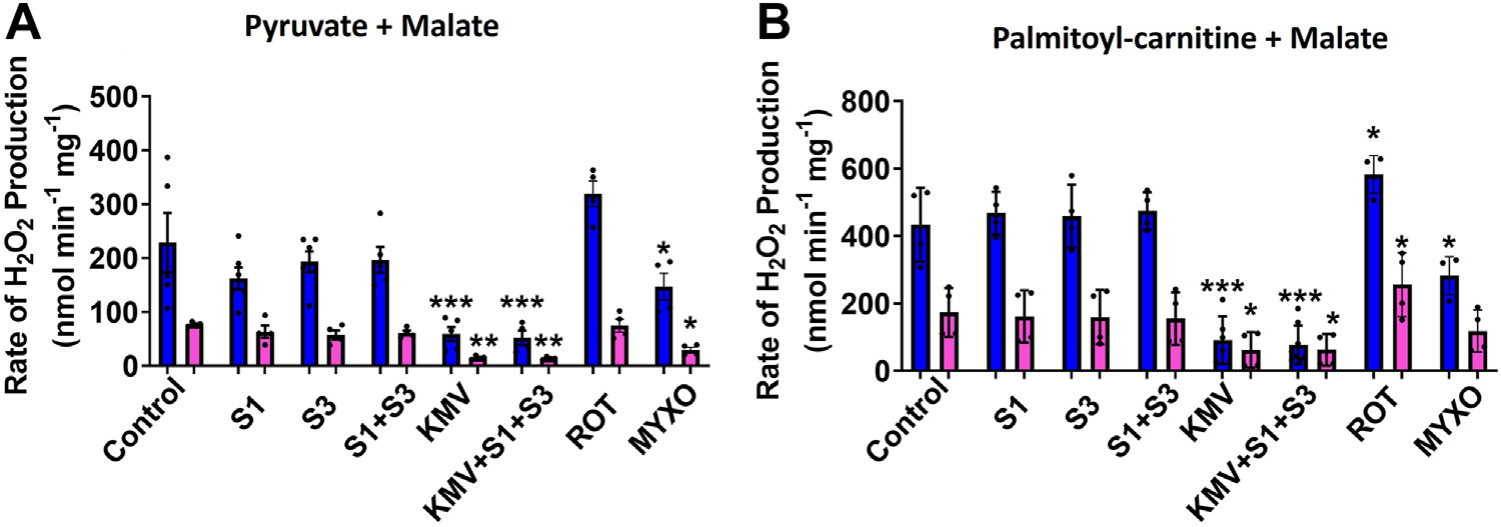
**KMV almost abolishes mH**_**2**_**O**_**2**_**generation by male and female liver mitochondria whereas complex I and III inhibitors, S1QEL 1.1 (S1) and S3QEL 2 (S3), have little effect.** The rate of mH_2_O_2_ generation was measured in male and female liver mitochondria fueled with pyruvate and malate (*A*) or palmitoyl-carnitine and malate (*B*). Mitochondria were treated with combinations of KMV, S1 (complex I inhibitor), and/or S3 (complex III inhibitor) prior to measuring mH_2_O_2_ generation. S1 and S3 have been shown to inhibit ROS production by complex I and III without interfering with respiration. In certain cases, mitochondria were treated with only rotenone (complex I inhibitor) or myxothiazol (complex III inhibitor). n = 4, mean ± SD, one-way ANOVA with a post hoc Fisher’s LSD test. KMV, 2-keto-3-methylvaleric acid; mH_2_O_2_, mitochondrial hydrogen peroxide; ROS, reactive oxygen species.

Next, we sought to test the role of KGDH in serving as a main source of mH_2_O_2_ further by comparing the inhibitor KMV to the novel inhibitors for reactive oxygen species (ROS) production by complexes I and III, S1QEL 1.1 (S1), and S3QEL 2 (S3). Both S1 and S3 have been shown to be potent site-specific inhibitors for ROS production by complexes I and III that do not interfere with respiration (41, 42). We conducted these experiments using male and female liver mitochondria energized with pyruvate and malate (Fig. 10*A*) or palmitoyl-carnitine and malate (Fig. 10*B*). Prior to measurements, mitochondria were treated with S1, S3, and KMV, alone or in combination or with only rotenone or myxothiazol (Fig. 10, *A* and *B*). Surprisingly, S1 and S3, either alone or in combination, had little to no effect on the rates of generation in male or female samples oxidizing either pyruvate or palmitoyl-carnitine (Fig. 10, *A* and *B*). By contrast, KMV almost abolished mH_2_O_2_ generation by samples oxidizing either pyruvate or palmitoyl-carnitine (Fig. 10*A* or 10*B*). The addition of KMV to mixtures containing S1 and S3 also almost abolished mH_2_O_2_ production (Fig. 10, *A* and *B*). Rotenone did not induce any significant effects on mH_2_O_2_ generation in mitochondria-oxidizing pyruvate (Fig. 10*A*) but did elevate it slightly in samples energized with palmitoyl-carnitine (Fig. 10*B*). Myxothiazol suppressed mH_2_O_2_ generation in male and female liver mitochondria–oxidizing pyruvate or palmitoyl-carnitine but not to the same extent as KMV (Fig. 10, *A* and *B*). We also examined if KMV could interfere with mH_2_O_2_ generation during the oxidation of palmitoyl-carnitine but when malate was excluded from reaction mixtures. As shown in Fig. S2, KMV did not affect the rate of mH_2_O_2_ generation when male and female liver mitochondria were oxidizing palmitoyl-carnitine in the absence of malate. In aggregate, these findings demonstrate KGDH, and not the ETC, is the main source of mH_2_O_2_ in liver mitochondria from male and female mice metabolizing pyruvate or palmitoyl-carnitine, but only when malate is used to prime the Krebs cycle.

### KMV is specific for KGDH and does not interfere with HADH, PDH, or the oxidation of succinate

**Figure 11:**
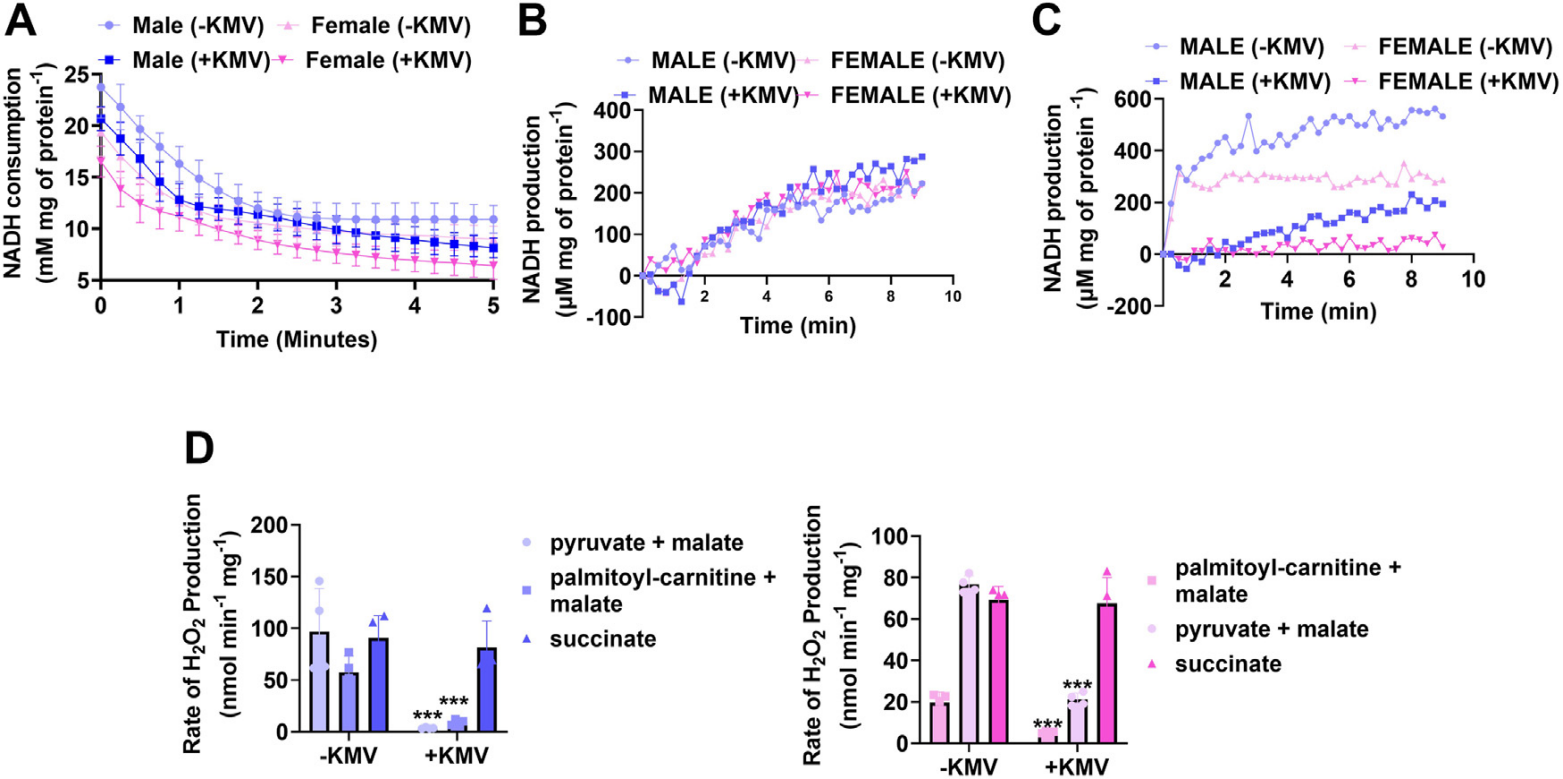
**KMV is a specific inhibitor for KGDH and does not interfere with fatty acid oxidation enzyme, β-hydroxy acyl-CoA dehydrogenase, pyruvate dehydrogenase, or inhibit mH**_**2**_**O**_**2**_**generation when succinate is oxidized by complex II.***A*, measurement of NADH consumption by HADH in permeabilized male and female liver mitochondria and the effect of KMV on the reaction. HADH was measured by assessing the consumption of NADH in the presence of acetoacetyl-CoA. n = 4, mean ± SD. *B*, measurement of the impact of KMV on the activity of PDH in permeabilized male and female liver mitochondria. n = 4, mean ± SD. *C*, measurement of the impact of KMV on the activity of KGDH in permeabilized male and female liver mitochondria. n = 4, mean ± SD. *D*, the rate of mH_2_O_2_ generation by male and female liver mitochondria energized with pyruvate and malate, palmitoyl-carnitine and malate, or succinate and treated with or without KMV. n = 4, mean ± SD, one-way ANOVA with a post hoc Fisher’s LSD test. KMV, 2-keto-3-methylvaleric acid; mH_2_O_2_, mitochondrial hydrogen peroxide.

KMV is an α-keto acid, structurally analogous to α-ketoglutarate. FAO requires the activity of HADH, which produces β-keto acyl-CoA for thiolation by CoASH. KMV has some structural properties resembling β-hydroxy acyl-CoA. So, to eliminate the possibility that KMV is interfering with FAO, we examined its effect on HADH. Liver mitochondria from male and female mice were permeabilized, incubated in KMV, and then HADH was measured by tracking the consumption of NADH in the presence of acetoacetyl-CoA. Incubation of permeabilized male and female liver mitochondria in KMV did not interfere with the NADH-consuming activity of HADH (Fig. 11*A*). Next, we wanted to verify the KMV was not inhibiting PDH and was specific for KGDH. The KMV did not disrupt the NADH generating capacity of PDH in permeabilized liver mitochondria from male and female mice (Fig. 11*B*). By contrast, the KMV abolished NADH production by KGDH in the permeabilized liver mitochondria collected from male and female mice (Fig. 11*C*).

We also wanted to verify that KMV was not abrogating mH_2_O_2_ generation by the ETC and the effect we were observing was strictly related to its site-specific inhibition of KGDH. To do this, liver mitochondria were incubated in KMV and then energized with pyruvate and malate, palmitoyl-carnitine and malate, or succinate. The rate of mH_2_O_2_ generation under these conditions was then determined. The expectation was the KMV would not impede mH_2_O_2_ generation fueled by succinate since it donates electrons directly to the ETC through complex II. KMV almost abolished mH_2_O_2_ production by isolated mitochondria from male and female mice oxidizing pyruvate and malate or palmitoyl-carnitine and malate (Fig. 11*D*). However, KMV had no effect on the rate of mH_2_O_2_ production when succinate was used to fuel the isolated liver mitochondria (Fig. 11*D*). Together, these findings demonstrate that KMV is site-specific for KGDH inhibition and does not interfere with PDH, FAO, or succinate-mediated mH_2_O_2_ generation by the ETC.

### KMV inhibits mH_2_O_2_ generation by mitochondria from male and female mice oxidizing short-, medium-, and long-chain fatty acids

**Figure 12:**
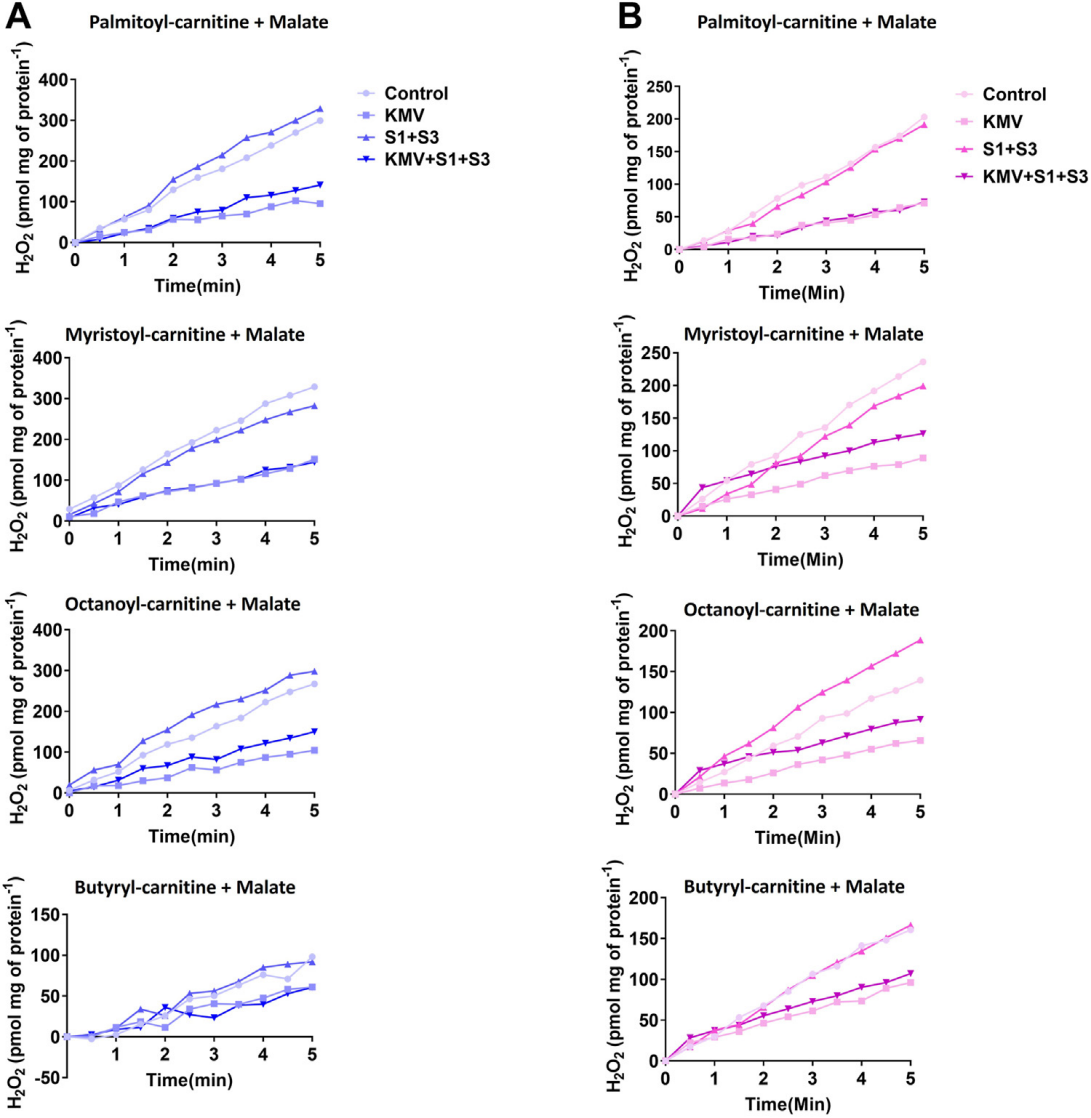
**KMV deactivates mH**_**2**_**O**_**2**_**generation by male and female liver mitochondria oxidizing long-chain (palmitoyl or myristoyl-carnitine), medium-chain (octanoyl-carnitine), short-chain (butyryl-carnitine) fatty acids.***A*, measurement of the rate of mH_2_O_2_ generation by male liver mitochondria oxidizing long, medium, and short-chained fatty acids with malate and in the presence or absence of KMV. n = 4, mean ± SD. *B*, measurement of the rate of mH_2_O_2_ generation by male liver mitochondria–oxidizing long-, medium-, and short-chained fatty acids with malate and in the presence or absence of KMV. n = 4, mean ± SD. KMV, 2-keto-3-methylvaleric acid; mH_2_O_2_, mitochondrial hydrogen peroxide.

We next sought to confirm KMV was inhibiting FAO-triggered mH_2_O_2_ generation through KGDH using mitochondria from male and female mice oxidizing butyryl-, octanoyl-, or myristoyl-carnitine in the presence of malate. Experiments were also carried out with palmitoyl-carnitine and malate. We also designed the experiments to include the inhibitors for complexes I and III of the ETC, S1, and S3. In the liver mitochondria from the males, the combination of S1 and S3 had little effect on mH_2_O_2_ generation by mitochondria fueled with palmitoyl-, myristoyl-, octanoyl-, or butyryl-carnitine, respectively (Fig. 12*A*). Inclusion of KMV alone abrogated mH_2_O_2_ generation in the male liver mitochondria–oxidizing palmitoyl-, myristoyl-, or octanoyl-carnitine (Fig. 12*A*). An inhibition was also observed in male liver samples oxidizing butyryl-carnitine, but the effect was less pronounced (Fig. 12*A*). We attribute this to the short length of the butyryl-carnitine carbon chain, which may make it a poor substrate for mH_2_O_2_ generation. Additionally, adding KMV to reaction mixtures containing S1 and S3 also interfered with mH_2_O_2_ generation, particularly when palmitoyl-, myristoyl-, or octanoyl-carnitine served as substrates (Fig. 12*A*). We generated similar findings with liver mitochondria isolated from female mice (Fig. 12*B*). S1 and S3 incubations had no impact on the rate of mH_2_O_2_ generation when palmitoyl-, myristoyl-, octanoyl-, or butyryl-carnitine were fueling mitochondria in the presence of malate (Fig. 12*B*). Including KMV in the mixtures with S1 and S3 inhibited the generation of mH_2_O_2_ in all four reaction mixtures (Fig. 12*B*). Additionally, KMV alone also abrogated the production (Fig. 12*B*).

## Discussion

The convention that complexes I and III of the ETC are the primary mH_2_O_2_ sources in mammalian mitochondria was challenged 2 decades ago when KGDH was identified as an important source (25, 26, 27). KGDH was originally found to support mH_2_O_2_ production during α-ketoglutarate oxidation in neuronal mitochondria, synaptosomes, and when purified from porcine heart (25, 26, 27). Later efforts revealed KGDH displayed a mH_2_O_2_ production rate eight times greater than that of complex I in rat skeletal muscles (28). After that, KGDH was shown to be a main source during pyruvate, lactate, and α-ketoglutarate oxidation in liver mitochondria (31, 32, 40). mH_2_O_2_ is vital for maintaining optimal liver health. In addition, the over generation of mH_2_O_2_ by liver mitochondria triggers NAFLD through the induction of oxidative distress (43). Identifying the main mH_2_O_2_ source in hepatic mitochondria could provide new opportunities for the development of therapeutic approaches for treating liver diseases like NAFLD. Sex is also a powerful factor that determines the onset and progression of NAFLD (44). Thus, the effect of sex on mH_2_O_2_ generation by individual sources in hepatic mitochondria must also be carefully considered when studying liver physiology and disease. In this study, we sought to characterize the effect of sex on the relative individual rates of mH_2_O_2_ generation by KGDH and components of the ETC, principally complexes I and III. We based this investigation on our previous findings showing KGDH in liver mitochondria is an important generator and that sex may be an important determinant for dictating the rate of mH_2_O_2_ production by this site. We also have a general interest in decoding the effect of sex on the individual rates of mH_2_O_2_ generation by the 12 sources of mitochondria (reviewed here (9, 39)). In the present study, we uncovered there is a sex effect on mH_2_O_2_ generation by the ETC, which we found to be substrate dependent. Complex I is a mH_2_O_2_ generator during RET from succinate in male but not female liver mitochondria. By contrast, complex III is a mH_2_O_2_ source in female liver mitochondria–oxidizing succinate. Similarly, complex I is the ETC source of mH_2_O_2_ in female liver mitochondria metabolizing palmitoyl-carnitine, whereas complex III is the supplier in male samples. The unexpected finding this study was the observation KGDH is the main mH_2_O_2_ generator in liver mitochondria from male and female mice oxidizing acyl-carnitines. Using various substrate and inhibitor combinations, we showed KGDH, not complexes I or III, is the main source of mH_2_O_2_ generation during FAO in liver mitochondria. These findings have strong implications for understanding: 1) how mitochondria generate mH_2_O_2_, 2) the effect of sex on the individual sources of mH_2_O_2_ production in mitochondria, 3) how mitochondria can potentially use generators outside of complexes I and III for eustress signaling in maintaining optimal hepatic health, and 4) how deregulated mH_2_O_2_ generation by KGDH during FAO may contribute to the development of hepatic diseases like NAFLD and non-alcoholic steatohepatitis.

### Increased coupling efficiency in OxPhos correlates with the lower rates for mH_2_O_2_ generation by female liver mitochondria

Mitochondria display many sex dimorphisms in bioenergetics and redox homeodynamics due to differences in gene expression, protein regulation, and epigenetic coding. Confirmed in this study, female liver mitochondria generally produce less mH_2_O_2_ when compared to males (45). Mitochondria contain 12 mH_2_O_2_ sources subdivided into two isopotential groups. These sources are flavin-dependent dehydrogenases and components of the ETC (14). Few studies have investigated the sex effects on these individual sources and their overall contributions toward total mH_2_O_2_ generation. Using different substrate/inhibitor combinations and gene knockout mouse models, we previously showed PDH and KGDH display rates of mH_2_O_2_ generation in male liver mitochondria that are much greater than samples collected from female mice (33, 46). This was also the case for DHODH, an oxidoreductase embedded in the mitochondrial inner membrane required for pyrimidine biosynthesis (47). In the present study, we found mH_2_O_2_ generation rates were significantly lower in female liver mitochondria than males during pyruvate, succinate, and palmitoyl-carnitine metabolism. The decreased rate of mH_2_O_2_ generation by the female liver mitochondria has been attributed to greater redox buffering capacity due to the higher concentration of antioxidant defenses and activation of mitochondrial mechanisms that suppress ROS production. Indeed, the concentration of GSH and its corresponding peroxidases (*e.g.,* Gpx1) is greater in female liver mitochondria and hepatocytes, which increases mH_2_O_2_ buffering capacity (48, 49). Liver mitochondria are more abundant in female rodents, have greater proton leaks, contain a higher mitochondrial DNA copy number and are richer in cardiolipin—factors that increase tolerance toward oxidative distress (50, 51). This greater capacity for mH_2_O_2_ elimination accounts for the lower rates of production we observed in our study. However, we also found this sex difference was also related to the better coupling efficiency of OxPhos in the female liver mitochondria, which suppressed mH_2_O_2_ generation. Female liver mitochondria displayed greater state 3 and state 3_U_ OCR, implying more electrons yielded from nutrient metabolism are successfully delivered to complex IV during OxPhos. This would limit the over-reduction of redox centers in the ETC and Krebs cycle enzymes, lowering the number of electrons available to generate mH_2_O_2_. The increased OxPhos efficiency in the female liver mitochondria was related to the augmentation of PDH, KGDH, and complex I expression, indicating improved coupling between nutrient oxidation and ATP production is associated with increased expression of the components required for mitochondrial metabolism and electron conductance.

### KGDH as a main source of mH_2_O_2_, even during FAO

**Figure 13:**
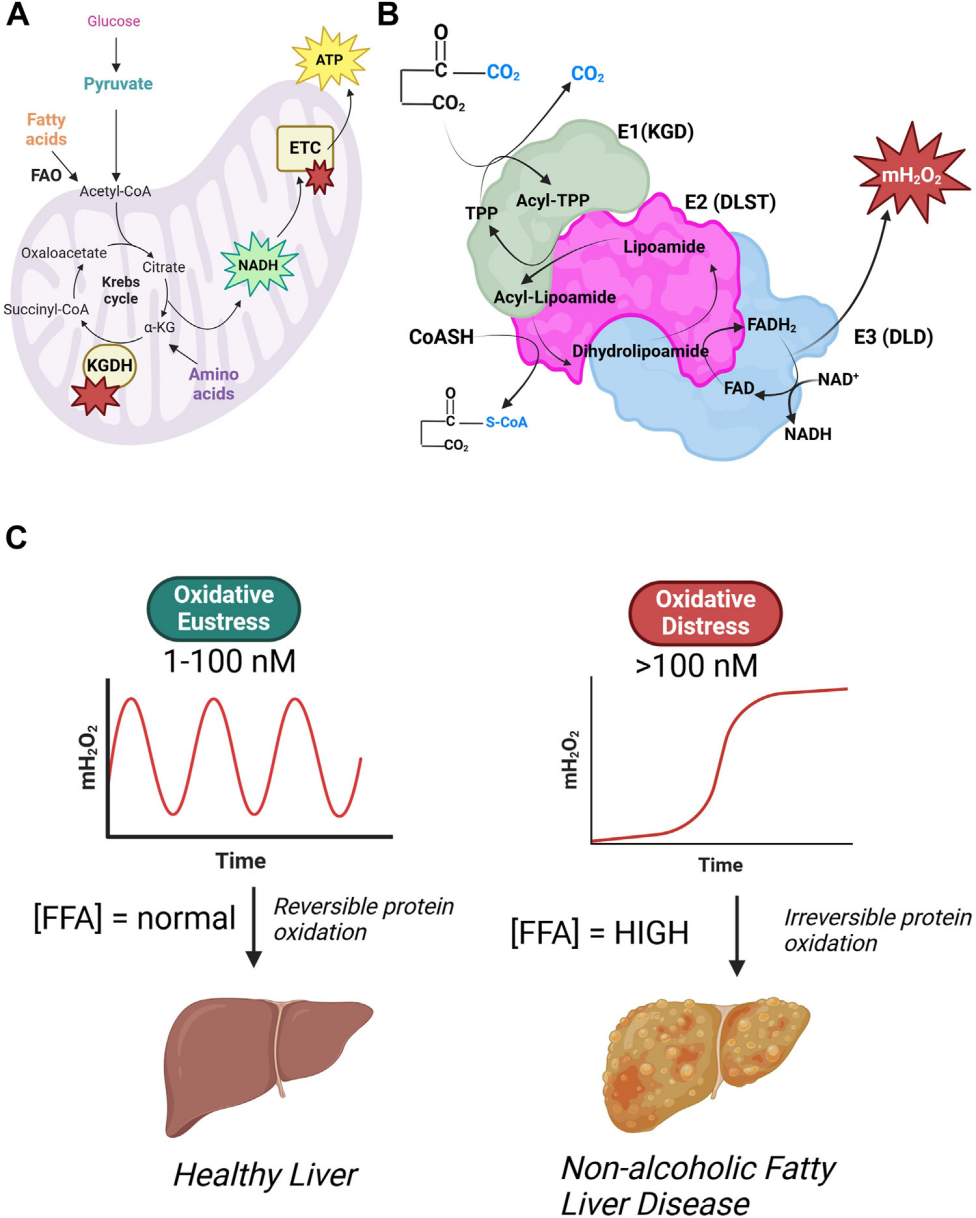
**A schematic view of how free fatty acids drive mH**_**2**_**O**_**2**_**by KGDH and the (patho)physiological consequences associated with the pathway.***A*, entry of pyruvate from monosaccharides (glucose) and FFAs into the acetyl-CoA pool of mitochondria. FFA degradation is driven by fatty acid oxidation (FAO). The acetyl-CoA condenses with oxaloacetate to form citrate which is then metabolized to α-ketoglutarate (αKG). KGDH oxidizes the αKG to succinyl-CoA. NADH formed by the Krebs cycle is used to power oxidative phosphorylation by injecting electrons into the electron transport chain (ETC). Both KGDH and the ETC are mH_2_O_2_ sources (*red star*), but KGDH is a more potent generator during hepatic fat metabolism. *B*, the basic structure of KGDH and the enzymatic steps that drive αKG decarboxylation by the E1 (αKG decarboxylase or KGD), lipoamide acylation and oxidation in the E2 subunit to form succinyl-CoA (dihydrolipoamide succinyltransferase or DLST), and the redox cycling of the FAD in the E3 subunit (dihydrolipoamide dehydrogenase or DLD), which forms NADH. The successful reduction of NAD by the E3 subunit requires the activity of all three subunits. mH_2_O_2_ is formed by the E3 subunit. *C*, the mH_2_O_2_ formed by KGDH during FAO could be used under normal physiological conditions to trigger oxidative eustress pathways. These pathways are tightly regulated and characterized by spatiotemporal increases and decreases in mH_2_O_2_ availability. Activating eustress through KGDH may be required for maintaining optimal hepatic health. Lipotoxicity caused by FFA overload has the opposite effect, triggering oxidative distress through the over generation of mH_2_O_2_ by KGDH . This causes nonalcoholic fatty liver disease (NAFLD). FFA, free fatty acids; KGDH, α-ketoglutarate dehydrogenase; mH_2_O_2_, mitochondrial hydrogen peroxide.

The α-keto acid dehydrogenase family of enzymes are the highest capacity mH_2_O_2_ sources in the NADH/NAD^+^ isopotential group (12). Of these four, KGDH displays the greatest capacity for mH_2_O_2_ generation. What remains unknown is why it produces more mH_2_O_2_ in comparison to the other α-keto acid dehydrogenase family members. Indeed, all four family members have the same basic structure and catalytic cycle (Fig. 13). The enzymes are composed of E1 (α-keto acid decarboxylase), E2 (dihydrolipoamide acyl transferase), and E3 (dihydrolipoamide dehydrogenase; dihydrolipoamide dehydrogenase) subunits (Fig. 13) (52). All four also share the same catalytic mechanism, relying on successive acyl group and electron transfers through the E1 to E3 subunits for the generation of acyl-CoA and NADH (Fig. 13) (39, 53). Additionally, the enzymes all generate mH_2_O_2_ through the E3 subunit and can potentially produce superoxide (O_2_^•-^) through a thiamine radical intermediate in the E1 component (54, 55). It has been postulated that the differences in mH_2_O_2_ generation may be related to the multimer composition of the α-ketoacid dehydrogenases (39). For example, mammalian PDH is a multisubunit holoenzyme composed of multiple copies of the E1, E2, and E3 subunits in a stoichiometry of 40:40:20 (56). By contrast, KGDH is predicted to be a ∼3.2 MDa complex made of 12 E1 and 12 E3 subunits that surround a 24-mer E2 subunit complex (57). Both PDH and KGDH also bind to distinct accessory proteins, are regulated through phosphorylation, which is the case for PDH, are distinctly controlled through allosteric regulation and associate with other redox enzymes, like NADPH producing nicotinamide nucleotide transhydrogenase, which can influence the overall rate of mH_2_O_2_. Both PDH and KGDH are also distinct in terms of their redox regulation by S-nitrosylation and S-glutathionylation. Collectively, these factors can be key determinants for the superior mH_2_O_2_ generation observed with KGDH.

KGDH also occupies a vital position in mitochondrial energy metabolism since it connects the oxidation of amino acids and acetyl groups derived from glycolysis, lactate, FAO, and ketone bodies in the Krebs cycle to the formation of ATP by OxPhos (Fig. 13). KGDH also catalyzes one of two high energy irreversible steps in the Krebs cycle, which relies on the successful transfer of electrons through an FAD prosthetic group to NAD in the E3 subunit. It is therefore probable that KGDH serves as a main source of mH_2_O_2_ and produces more than the other α-keto acid dehydrogenases because it is a point of convergence for the catabolism of several types of fuel by the Krebs cycle. Previous studies that have examined mH_2_O_2_ generation by KGDH have almost exclusively studied it using intact or permeabilized mitochondria energized with pyruvate or glutamate in combination with malate or just α-ketoglutarate. These experimental approaches, which were described above, led to the discovery that KGDH is an important source of mH_2_O_2_. Here, we have confirmed KGDH is an important source of mH_2_O_2_ in liver mitochondria from male and female mice oxidizing pyruvate with malate. However, our main discovery was that KGDH is also the major point for mH_2_O_2_ genesis by KGDH during FAO. Indeed, to our surprise, the rate of mH_2_O_2_ production during FAO in male and female liver mitochondria could almost be abolished by KMV. Importantly, the KMV did not interfere with FAO enzyme, HADH, did not cross react with PDH and did not inhibit succinate metabolism. By contrast, the KMV abrogated KGDH showing it is a site-specific inhibitor for this α-keto acid dehydrogenase. However, the most notable observations we made when exploring the mH_2_O_2_ producing potential of KGDH during FAO in the male and female liver mitochondria are as follows: 1) malate is required to induce this mH_2_O_2_ generation, 2) S1 and S3 inhibitors of ROS generation by complexes I and III had little effect on mH_2_O_2_ production during the metabolism of short-, medium-, and long-chain acyl-carnitines or pyruvate, 3) KMV, either alone or in combination with S1 and S3, almost abolished mH_2_O_2_ generation by KGDH in mitochondria oxidizing short-, medium-, and long-chain acyl-carnitines or pyruvate, and 4) the KMV abolished generation in both male and female samples, suggesting KGDH is a main source of mH_2_O_2_ regardless of sex.

### Is KGDH a main mH_2_O_2_ source in oxidative eustress and oxidative distress?

The observation 2 decades ago that KGDH can supply mH_2_O_2_ in neural tissue was a major finding since it showed sources outside of the ETC could also be important generators (25, 26, 27, 58). At the time, it was aptly proposed KGDH could be a source of oxidative distress causing cell disease and death, especially when NADH oxidation by complex I was deficient (27, 59, 60, 61). It was then found KGDH and PDH display greater relative individual rates of mH_2_O_2_ production than complex I (28). Notably, the same group reported BCKDH, another member of the α-keto acid dehydrogenase family of enzymes that is required for branched chain amino acid metabolism, also produces more mH_2_O_2_ when compared to complex I (28). This same group then reported OADH, a fourth α-keto acid dehydrogenase in mitochondria, also generates more mH_2_O_2_ than complex I (30). Similarly, KGDH, and to a lesser extent PDH and BCKDH, display rates of mH_2_O_2_ generation that are greater than that of complex I in mouse liver mitochondria (31, 32, 33). Additionally, KGDH and PDH can also produce mH_2_O_2_ by RET from NADH (26, 36, 37).

The findings we have made here show KGDH is a main mH_2_O_2_ supplier in liver mitochondria and is an important source during FAO. These findings have strong implications for understanding the role mH_2_O_2_ genesis by KGDH plays in hepatic health and disease. NAFLD is surging due to the increased preponderance of metabolic diseases and has a wide spectrum of clinical manifestations ranging from simple steatosis to more severe forms like cirrhosis (62, 63). A distinguishing feature of NAFLD is intrahepatic lipotoxicity triggered by free fatty acid (FFA) and acyl-carnitine overload, which results in the over generation of mH_2_O_2_ and the induction of oxidative distress (62, 64, 65, 66, 67). Notably, the source of this mH_2_O_2_ in the progression of NAFLD and the manifestation of its more severe forms to nonalcoholic steatohepatitis, cirrhosis, and hepatocellular carcinoma is poorly defined. Difficulties in identifying the key source may have been related to assuming complexes I and III are the main mH_2_O_2_ suppliers. However, one study published by Seifert *et al*. did suggest ETC-independent sources also produce mH_2_O_2_ during acyl-carnitine metabolism (68). Our findings show that this unidentified source of mH_2_O_2_ formed during FAO is KGDH. It is important to emphasize here that we did not investigate relationships between KGDH activity, FFA-mediated mH_2_O_2_ generation, and the onset of NAFLD and its more severe forms. However, we do supply convincing evidence showing that KGDH is the main mH_2_O_2_ source when hepatocytes are overloaded with fat.

Another key observation we made here is KGDH was a main mH_2_O_2_ generator in male and female liver mitochondria metabolizing acyl-carnitine. NAFLD has a higher preponderance in men than in premenopausal women (69). However, NAFLD surges in postmenopausal women, which parallel the onset of obesity, type 2 diabetes mellitus, and other disorders (70). The sex dimorphic effects on NAFLD development have been related to superior mitochondrial redox poise, mH_2_O_2_ budgeting, and fuel metabolism in fertile female rodents and premenopausal women (45, 49, 50, 69, 70, 71). NAFLD is accelerated in ovariectomized rodents and postmenopausal women due to mitochondrial defects, causing oxidative distress (70, 72). Taken together with our findings, KGDH likely serves as this mH_2_O_2_ source that triggers oxidative distress and rapidly accelerates the onset of NAFLD in ovariectomized rodents and postmenopausal women. Although speculative, targeting KGDH to modulate the rate of its mH_2_O_2_ release may serve as a new way to treat and prevent NAFLD in men and postmenopausal women.

## Experimental procedures

### Animals and preparation of mouse liver mitochondria

Animals were cared for in accordance with the principles and guidelines of the Canadian Council on Animal Care and the Institute of Laboratory Animal Resources. Animal experiments were approved by the Facilities Animal Care Committee in the Faculty of Agricultural and Environmental Sciences at McGill University. Wild-type male and female C57BL/6N mice aged 9 weeks were purchased from Charles River Laboratories. They were housed at 25 °C on a 12-h day/night light cycle and provided unlimited access to water and standard chow (Inotiv TD2020X).

Liver mitochondria were isolated from mice ranging from 10 to 13 weeks of age. This procedure does not compromise the inner mitochondrial membrane integrity or alter the overall ultrastructure of the cristae (73, 74). Mice were euthanized *via* cervical dislocation while under 5% isoflurane. All steps of the isolation were performed on ice or at 4 °C. Livers were surgically removed and placed in ice-cold MESH buffer (220 mM mannitol, 1 mM EGTA, 70 mM sucrose, 10 mM Hepes, pH 7.4). Mouse livers were cut into pieces and washed in ice-cold MESH buffer to remove excess blood and fur. Livers were minced using a razor on a Teflon watch glass on ice and homogenized in 25 ml of MESH supplemented with 0.5% w/v defatted bovine serum albumin using Teflon pestles and a Glas-Col variable speed homogenizer. Homogenates were centrifuged at 900*g* and 4 °C for 9 min to pellet cellular debris and nuclei (Sorvall Lynx 4000). The supernatant was collected and centrifuged at 12,000*g* and 4 °C for 9 min to pellet the mitochondria. The pellet was washed and resuspended in 500 μl of MESH. Protein equivalents were determined using a Bradford assay. BSA was used to construct the calibration curve.

### Interrogating mH_2_O_2_ production in liver mitochondria

The rate of mH_2_O_2_ production by male and female WT C57BL/6N mouse liver was measured *via* Amplex UltraRed (AUR) assay and our established substrate/inhibitor toolkit (reviewed in (9)). Isolated mitochondria were diluted to 5 mg/ml in MESH and stored on ice prior to running the assays. Samples were then diluted to a final concentration of 0.5 mg/ml in MESH in the wells of a 96-well black plate. The final reaction volume was 200 μl. The mitochondria were incubated for 5 min to equilibrate at 25 °C and then treated with the following substrate/inhibitor combinations: 1) 5 mM succinate with 0 to 10 mM malonate (complex II inhibitor), 2) 5 mM succinate with 1 μM rotenone and 0–10 μM myxothiazol, 3) 5 mM pyruvate and 2 mM malate with 0–10 mM 2-keto-3-methylvaleric acid (KMV), 4) 100 μM palmitoyl-carnitine and 2 mM malate with 1 μM rotenone and 0–10 μM myxothiazol, and 5) 100 μM palmitoyl-carnitine and 2 mM malate with 1 μM rotenone, 5 μΜ myxothiazol, and 0 to 10 mM KMV. In certain cases, only palmitoyl-carnitine, myristoyl-carnitine, octanoyl-carnitine, or butyryl-carnitine (each 100 μM), or 5 mM pyruvate with 2 mM malate or 5 mM succinate were added with 10 mM KMV, 4 μM rotenone, or 40 μM myxothiazol. A full breakdown of the sites of action for the different substrate/inhibitor combinations is supplied in Figure 1. The reactions were then supplemented with 3 U/ml horseradish peroxidase, 25 U/ml superoxide dismutase, and 20 μM AUR (all final concentrations). The conversion of AUR to fluorescent resorufin was measured every 30 s for 5 min at 565/600 nm using a Cytation 5 microplate reader equipped with Gen 5 3.11 software. mH_2_O_2_ production was normalized to mitochondrial protein equivalents and background fluorescence. The rate of mH_2_O_2_ production was calculated using a linear calibration curve constructed using 1 to 1000 nM H_2_O_2_ and the AUR reagents.

### Seahorse XFe24 assays

Examination of mitochondrial bioenergetics using the Agilent Seahorse XFe24 analyzer (XFe24) was performed as described previously (74). Briefly, mitochondria were isolated from livers as described above with the addition of 10 mM pyruvate and 2 mM malate in the MESH. Samples were diluted to 0.2 mg/ml and 50 μl added to the wells of a XFe24 tissue culture plate. The plate was then centrifuged at 1200*g* for 20 min at room temperature in a Sorvall X Pro Series swing bucket centrifuge. Plate wells were then supplemented with 450 μl of respiration buffer (MESH supplemented with 10 mM KH_2_PO_4_, 2 mM MgCl_2_, and 0.1% (w/v) defatted BSA. Respiration buffers contained 10 mM pyruvate and 2 mM malate, 5 mM succinate, or 100 μM palmitoyl-carnitine and 2 mM malate. Mitochondria were incubated in respiration buffer for 30 min at 37 °C. Injection protocols were developed using Wave Controller Software 2.4. Mitochondrial bioenergetics was interrogated by first measuring state 4 respiration (substrates alone with mitochondria). State 3, state 4_O_ (oligomycin), and state 3_U_ (uncoupled) were induced by injecting ADP (4 mM final concentration), oligomycin (2.5 μg/ml final concentration), and FCCP (4 μM final concentration), respectively. State 4: conditions when mitochondria are exposed to only the substrate(s), state 3: the proton circuit is completed by the addition of ADP, state 4_O_: ATP synthase is arrested with oligomycin, and state 3_U_: max respiration induced by the protonophore FCCP. Antimycin A (4 μM) was injected at the end of the protocol to arrest the ETC. All oxygen consumption rates (OCRs) were normalized to mitochondrial content and rates of O_2_ consumption after the antimycin A injection.

### β-hydroxyacyl-CoA dehydrogenase assay

β-hydroxyacyl-CoA dehydrogenase (HADH) was assayed using permeabilized mitochondria. Mitochondria were diluted to 1 mg/ml in MESH containing 0.1% Triton X-100 and then incubated on ice for 60 min. Samples were mixed every 10 min during the 60-min incubation. Samples were diluted to a final concentration of 0.1 mg/ml in MESH-B. The samples were first equilibrated for 5 min at room temperature and then incubated for 15 min in 10 mM KMV. 0.1 mM acetoacetyl-CoA, 0.3 mM thiamine pyrophosphate, and 0.1 mM NADH were then added for a final reaction volume of 200 μl. HADH activity was estimated by measuring NADH consumption at 340 nm. The enzyme’s activity was calculated using a molar extinction coefficient of ε_340_ = 6220 M^−1^ cm^−1^ and the Beer-Lambert Law.

### PDH and KGDH activity assays

PDH and KGDH activities were assayed using permeabilized mitochondria as in the HADH assay. Samples were diluted to a final concentration of 0.1 mg/ml in MESH-B and incubated in 10 mM KMV. 0.1 mM CoASH, 0.3 mM thiamine pyrophosphate, 0.1 mM NAD^+^, and either pyruvate or α-ketoglutarate (0.1 mM) were then added. PDH and KGDH activity was estimated by measuring NADH production at 340 nm and the molar extinction coefficient described above.

### Immunoblot

Mitochondria were diluted to 2 mg/ml in 1× Laemmli buffer and 1× RIPA solution and incubated for 10 min at 100 °C. Twenty microliters of sample was loaded in each well, and proteins were electrophoresed through a 10% isocratic denaturing acrylamide gel. Proteins were then electroblotted to nitrocellulose membranes by tank transfer. Successful transfer was confirmed by Ponceau S staining. Membranes were blocked for 1 h with tris-buffered saline with 0.1% Tween-20 containing 5% (w/v) nonfat skim milk and incubated overnight in primary antibodies directed against PDH (Abcam, PDH cocktail), KGDH (Abcam, E1 subunit), and the respiratory chain (Abcam, OxPhos cocktail). NDUFS1 served as a loading control for blots probed for PDH and KGDH. In these cases, membranes were cut in between the approximate molecular weights of NDUFS1 (∼75 KDa) and the E2 subunit for PDH (highest MW for all three subunits at ∼68 KDa) or the E2 subunit for KGDH (∼100 KDa) with a razor. Membranes were cut at the Ponceau S stain stage of the blot, and molecular weights were estimated based on the migration of the prestained molecular standard (PageRuler Prestained Molecular Standard; Thermo Fisher Scientific). In one case, the Ponceau S stain was used as the loading control for the blot because the molecular weight of the loading control, NDUFS1, has a very similar molecular weight to a subunit of interest in the OxPhos antibody cocktail. Membranes were then washed and probed with goat anti-mouse and anti-rabbit antibodies tagged with horseradish peroxidase (Abcam). Activity bands were visualized with chemiluminescent substrate (Thermo Fisher Scientific) and imaged using a Li-Cor C-Digit Scanner. Bands were quantified using ImageJ (https://imagej.net/ij/download.html) software.

### Statistical analysis

All experiments were conducted on mitochondria isolated from only one liver surgically removed from male and female mice. Rates of mH_2_O_2_ generation by liver mitochondria, enzyme activities, and XFe24 data were processed in Microsoft Excel. Rate results and traces were then collated into GraphPad Prism 9 (https://www.graphpad.com/) for analyses. XFe24 assays were performed in quadruplicate (four replicates per plate per sample) and on mitochondria isolated from the livers of three separate mice (N = 3). Immunoblots were conducted using liver mitochondria collected from three separate mice (N = 3). Blots were quantified using ImageJ and analyzed in GraphPad. mH_2_O_2_ generation assays and enzyme activities were conducted in duplicate (two replicates per plate per sample) and on samples collected from four separate mice (N = 4). Rates for mH_2_O_2_ generation were estimated using standard curves. Results were analyzed by Student’s *t* test or one-way and two-way ANOVAs with a Fisher's least significant square post hoc test. ∗, # = *p* ≤ 0.05, ∗∗, ## = *p* ≤ 0.01, ∗∗∗, ### = *p* ≤ 0.005, and ∗∗∗∗, #### = *p* ≤ 0.001. Figure 1 was generated using BioRender software (https://app.biorender.com/user/signin). ∗ denotes a statistically significant change within a group and # denotes a sex effect. All values are presented as the mean ± SD.

## Conclusions

Taken together, we discovered the Krebs cycle can be a major source of mH_2_O_2_ during FAO (Fig. 13). We specifically identified KGDH as the source of this mH_2_O_2_. In addition to this, we present, for the first time, evidence that would suggest complex I and III of the ETC are not major mH_2_O_2_ suppliers during FAO. It is entirely possible that previous studies have overestimated the contributions of these ETC components toward the overall emission of mH_2_O_2_ into mammalian cells. Therefore, it is advised that KGDH (and other non-ETC sources) are considered when studying mitochondrial mH_2_O_2_ production in the contexts of cellular eustress and distress. Our findings also demonstrate some intriguing sex differences in mH_2_O_2_ generation. mH_2_O_2_ production, in general, was greater in samples collected from male mice, which is not a new observation regarding the study of sex effects in redox biology. However, what is novel is the finding mH_2_O_2_ production by complexes I and III display sex effects in rate of genesis, which we found was dependent on substrate preference. What was also highly notable was that KGDH was a main source of mH_2_O_2_ under all experimental conditions that included malate, showing the rate of production by KGDH is sex-independent. Another consideration is that KGDH, although very similar in basic structure to the other α-ketoacid dehydrogenases, seems to be unique in its mitochondrial redox role. This is because we found KGDH, not PDH, is a major mH_2_O_2_ source. We propose that KGDH is a main oxidative eustress platform in male and female hepatocytes for the maintenance of optimal liver health (Fig. 13). Additionally, we propose it is KGDH, not the ETC, that serves as a major source of ROS for the induction of FAO-mediated oxidative distress during lipotoxicity caused by FFA overload leading to the onset of NAFLD (Fig. 13).

## Limitations of the study

We relied on the use of isolated liver mitochondria to interrogate mH_2_O_2_ which loses the *in vivo* context and therefore diminishes the significance of our findings. This could have been mitigated using cell culture systems containing knockouts for the individual mH_2_O_2_ sources. Although cell culture systems are considered an *in vitro* model, the cellular environment is still intact giving the findings more physiological relevance. Also, although we examined the effect of KMV on HADH, we did not interrogate its impact on FAO. This could be done by ^14^C tracer studies. However, it should be noted the KMV did not affect succinate driven mH_2_O_2_ generation, which demonstrates it does not inhibit the ETC. The sex effects still require more clarification, and we did not consider other potentially important sources like BCKDH and OADH. Another limitation is that we conducted our study on mice fed a standard TD2020X diet from Inotiv. Mice were not fed control matched, high-fat, or high-sucrose/high-fat diets, which could provide valuable information on determining if KGDH is also a main source of mH_2_O_2_ in diet-induced obesity/NAFLD models. It is important to emphasize the main goal of the study presented here was to interrogate the mH_2_O_2_-generating potential of KGDH and complexes I and III when mitochondria are energized with acyl-carnitines. However, future studies should more strongly consider diet effects on the rate of mH_2_O_2_ generation by KGDH.

## Data availability

Data are available in the main text or the Supporting information.

## Supporting information

This article contains supporting information.

## Conflict of interest

The authors declare that they have no conflicts of interest with the contents of this article.
