# Supplementary material for: Fatty acid oxidation drives mitochondrial hydrogen peroxide production by α-ketoglutarate dehydrogenase
Source: J Biol Chem. 2024 Mar 11;300(4):107159. doi: 10.1016/j.jbc.2024.107159 (PMC10997840; doi:10.1016/j.jbc.2024.107159)
Supplement: Supporting Figures S1 and S2 [file mmc1.docx]

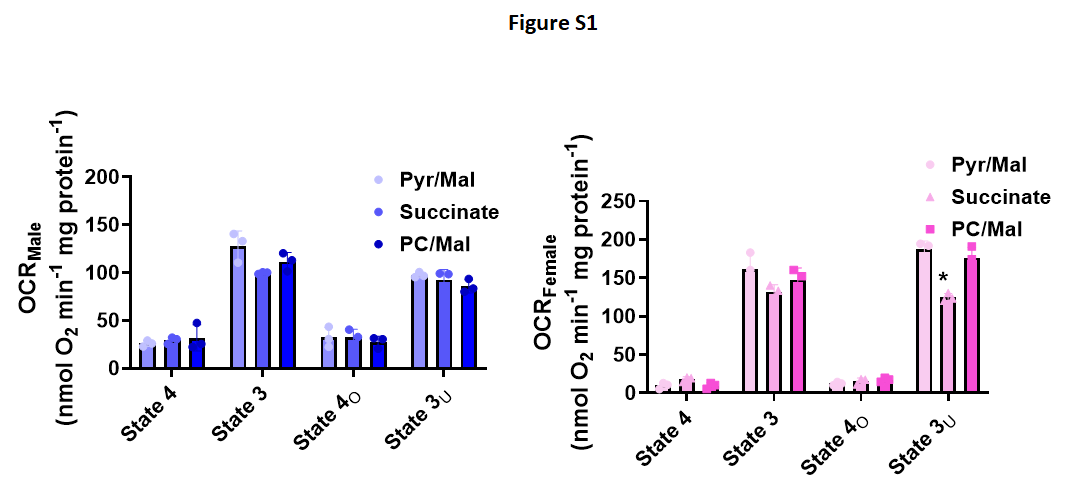


**Figure S1:** Comparison of OCR in male (left) and female (right) liver mitochondria fueled with pyruvate/malate (pyr/mal), succinate, or palmitoyl-carnitine/malate (PC/Mal). OCR was measured in the presence of substrates only first (state 4) followed by the injection of ADP, oligomycin, and FCCP to measure state 3, state 4_O_, and state 3_U_, respectively. All values were normalized to mitochondrial protein equivalents and the OCR measured after treatment with antimycin A (last injection of the assay). N=3, mean±SD.


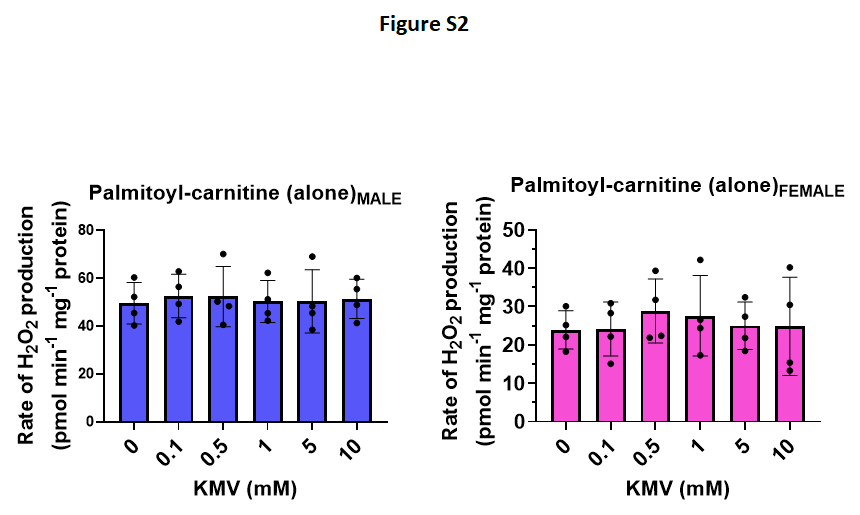


**Figure S2**: The effect of KMV on the rate of mH_2_O_2_ generation by male (left) and female (right) liver mitochondria oxidizing palmitoyl-carnitine, only. N=4, mean±SD.
